# Hydrocarbon-Contaminated Sites: Is There Something More Than *Exophiala xenobiotica*? New Insights into Black Fungal Diversity Using the Long Cold Incubation Method

**DOI:** 10.3390/jof7100817

**Published:** 2021-09-29

**Authors:** Daniela Isola, Alessandra Scano, Germano Orrù, Francesc Xavier Prenafeta-Boldú, Laura Zucconi

**Affiliations:** 1 Department of Ecological and Biological Sciences (DEB), University of Tuscia, 01100 Viterbo, Italy; zucconi@unitus.it; 2 Department of Surgical Sciences, University of Cagliari, 09124 Cagliari, Italy; alessandra.scano77@unica.it (A.S.); orru@unica.it (G.O.); 3 Program of Sustainability in Biosystems, Institute of Agrifood Research and Technology (IRTA), 08140 Barcelona, Spain; francesc.prenafeta@irta.cat

**Keywords:** black yeasts, diesel car tank, diesel gasoline fuel pump dispenser, *Knufia*, opportunistic *Exophiala* species, extreme environments, hydrocarbon bioremediation, fuel degraders, *Scolecobasidium*, volatile organic compounds (VOCs)

## Abstract

Human-made hydrocarbon-rich environments are important reservoirs of microorganisms with specific degrading abilities and pathogenic potential. In particular, black fungi are of great interest, but their presence in the environment is frequently underestimated because they are difficult to isolate. In the frame of a biodiversity study from fuel-contaminated sites involving 30 diesel car tanks and 112 fuel pump dispensers (52 diesel and 60 gasoline, respectively), a total of 181 black fungal strains were isolated. The long cold incubation (LCI) of water-suspended samples, followed by plating on Dichloran Rose Bengal Chloramphenicol Agar (DRBC), gave isolation yields up to six times (6.6) higher than those of direct plating on DRBC, and those of enrichment with a phenolic mix. The sequencing of ITS and LSU-rDNA confirmed the dominance of potentially pathogenic fungi from the family Herpotrichiellaceae and *Exophiala xenobiotica*. Moreover, other opportunistic species were found, including *E. opportunistica*, *E. oligosperma*, *E. phaeomuriformis*, and *Rhinocladiella similis*. The recurrent presence of *E. crusticola*, *Knufia epidermidis*, *Aureobasidium melanogenum*, *Cladosporium* spp., and *Scolecobasidium* spp. was also recorded. Interestingly, 12% of total isolates, corresponding to 50% of taxa found (16/32), represent new species. All the novel taxa in this study were isolated by LCI. These findings suggest that black fungal diversity in hydrocarbon-rich niches remains largely unexplored and that LCI can be an efficient tool for further investigations.

## 1. Introduction

Extreme environments are reservoirs for specialized microorganisms able to maintain their metabolic activity despite physical and chemical restrictive conditions. Black fungi is the epithet used since the 1990s to identify a group of polyextremotolerant melanized organisms adapted to hostile environments where they cope with several types of stresses including rapid changes in key environmental parameters [1,2]. For their outstanding morpho-ecological features, black fungi are also called black yeasts, rock-inhabiting fungi (RIF), black meristematic fungi, and microcolonial fungi (MCF) [3]. These fungi should not be confused with the emerging colloquially homonymous ‘black fungus’ from the order Mucorales, responsible for opportunistic infections associated with the COVID-19 pandemic [4]. Indeed, the poikilo-tolerant black fungi belong to two main lineages, namely Dothideomycetes and Eurotiomycetes [5,6]. The former are overrepresented in cold natural habitats, while the latter, with a few exceptions, thrive in hot, semi-arid climates [5]. Chaetothyrialean fungi are well known for their dualism, where the ability to assimilate alkylbenzenes (hydrocarbonoclastic activity) and the tendency toward being virulent represent the two sides of the same coin. This feature is particularly marked within the family Herpotrichiellaceae, including the causative agents of severe infections in humans, not limited to immunosuppressed hosts, and in cold-blooded animals with a preference towards the central nervous system [7,8,9,10]. Currently, a clear delimitation between hydrocarbonoclastic and pathogenic species is missing, since the known toluene-growing species from the genus *Exophiala*, such as *E. oligosperma*, *E. xenobiotica,* and *E. mesophila*, are recognized as biosafety level 2 (BSL2) microorganisms associated with opportunistic infections in humans [6,10].

Chaetothyrealean fungi have been isolated from several man-made environments that are generally grouped into two categories. The first category includes wet oligotrophic environments subjected to extreme temperatures and biocidal products (e.g., detergents) such as dishwashers, kitchen sinks [11,12,13,14,15,16], moist surfaces and wet cells in bathrooms [17,18,19,20,21], laundry machines [22,23,24], hospital environments [25], and public bathing facilities [26,27]. The second consists of niches related to petroleum and coal products, including gasoline car tanks, creosote-treated wood (e.g., railway sleepers), surfaces containing machinery oil, dirt, and hydrocarbon-polluted soils [14,24,28,29,30,31,32]. Due to their oligotrophic nature and low competitiveness under standard laboratory conditions, these fungi typically require specific isolation methods that control fast-growing microorganisms. Several protocols have been developed to isolate black fungi in relation to the type of sample and/or the desired fungal traits: (1) pre-incubation in acidic medium at high temperature to favor thermophilic species [33]; (2) extraction via mineral oil to select hydrophobic black fungal propagules [30,31]; and (3) enrichment on volatile aromatic hydrocarbons including toluene, styrene, or a mixture of phenolic compounds to isolate fungi with aromatic-degrading abilities [24,32,34,35,36].

Hydrocarbon-contaminated sites may harbor microorganisms with biodegradative potential and/or opportunistic/pathogenic traits [6,8,11,19,24,37]. As indicated by its epithet, *Exophiala xenobiotica* is a species frequently found in habitats rich in monoaromatic hydrocarbons and alkanes which can also cause opportunistic mycoses [37]. To increase our knowledge on hydrocarbonoclastic fungi and their associated potential biohazards during the handling of fuels, we focused on their presence in car fuel tank caps and fuel pump dispensers. Previous studies have been carried out with gasoline [24] but, to the best of our knowledge, the fungal colonization of diesel car tanks and fuel dispensers has never been investigated. The efficiency of the isolation methods can change with the sample type and/or the environmental matrix investigated. Therefore, the development of additional protocols under different conditions is of great significance. In this work, we tested the efficacy of three isolation protocols to deepen our knowledge on the biodiversity of black fungi able to thrive on fuel pump dispensers and diesel car tanks. The collected samples were processed using three different protocols: (1) direct plating, (2) enrichment with a phenolic mix, and (3) long incubation (six to eight months) of water suspended samples at a low temperature before plating. The last is a new protocol inspired by direct observation of low-temperature (1 ± 1 °C), long-stored sample suspension vials showing clear signs of black fungal growth on their walls [38].

The obtained isolates were identified by ITS and LSU rDNA sequencing, and black fungal diversity results were analyzed and discussed in terms of their ecophysiology and phylogeny.

## 2. Materials and Methods

### 2.1. Sampling

**Figure 1:**
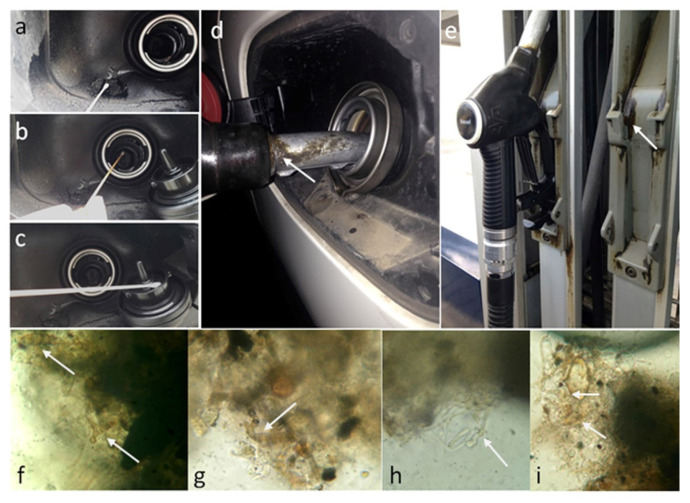
Sampled surfaces: (**a**–**c**) diesel car tanks; (**d**,**e**) fuel pumps dispenser. (**f**–**i**) Magnification of biofilms taken from dripping fuels; white arrows indicate thick dark hyphae.

Samples were collected between Jan and Mar 2014 using sterile cotton swabs. The investigated sites were 30 diesel car tanks (DCT), 52 diesel pump dispensers (DPD), and 60 gasoline pump dispensers (GPD). Only diesel cars ten years or older were considered for this study, since spore landing may primarily occur during refueling procedures, and colonies may take considerable time to develop [24]. Three samples were taken from each diesel car: (1) from the inner part, (2) from the external part of the tank fuel pouring hole, and (3) from the internal lid surface (Figure 1a–c). Only one sample was taken from each fuel pump dispenser where dripped fuel and dark deposits were visible (Figure 1d,e). Samples were collected from different geographical areas in central Italy.

### 2.2. Isolation Protocols

Sampling swabs were washed in 1.5 mL centrifuge tubes containing 1 mL of sterile distilled water (dH_2_O) and stored at 1 ± 1 °C until use. Sample suspensions were used for (1) direct plating (DP, 50 μL) on Dichloran Rose Bengal Chloramphenicol Agar (DRBC, Laboratorios Conda, Madrid, Spain); (2) inoculating an enrichment liquid medium containing a phenolic mix of 4-hydroxybenzoic acid, protocatechuic acid, phenylacetic acid, and catechol (0.125 g/L each) and plated in the same solid medium as described by Isola et al. [24] (phenolic enrichment, PE); and (3) a six to eight months long storing/incubation (long cold incubation, LCI) at low temperature (1 ± 1 °C) and plated on DRBC (50 μL). Plates were then incubated at room temperature (18 ± 1 °C) and inspected daily for two to three weeks. All morphologically distinct black colonies were transferred onto malt agar (MA, malt extract 30 g/L, agar 15 g/L) and processed for molecular identification. Isolated strains were deposited in the Culture Collection of Fungi from Extreme Environments (CCFEE) of the Tuscia University (Viterbo, Italy).

### 2.3. Molecular Identification

Genomic DNA was extracted using a Nucleospin Plant kit (Macherey-Nagel, Düren, Germany) following the protocol optimized for fungi. ITS amplifications were performed using BioMix (BioLine, Luckenwalde, Germany) in a total volume of 25 μL. In each reaction solution, 5 pmol of each primer (ITS4 and ITS5) and about 40 ng of template DNA were added. Amplifications were carried out using MyCycler™ Thermal Cycler (Bio-Rad Laboratories, Munich, Germany), applying the following protocol: initial denaturation step for 3 min at 95 °C, 35 cycles of 95 °C for 30 s, annealing at 55 °C for 30 s, extension at 72 °C for 32 s, followed by a final extension at 72 °C for 5 min [39]. When ITS matching was unfruitful, additional LSU amplifications (LR0R-LR7) were generated and analyzed to better define taxa boundaries. The applied amplification protocol was: initial denaturation step for 3 min at 95 °C, 35 cycles of denaturation at 95 °C for 45 s, annealing at 52 °C for 30 s, and extension at 72 °C for 2 min, with a 5 min final extension at 72 °C [39]. Sequences obtained from Macrogen Inc. (Madrid, Spain) were analyzed using ChromasPro v. 1.41 (Technelysium, Southport, Queensland, Australia).

Similarity searches were performed using BLASTn (NCBI, National Center for Biotechnological Information) excluding “uncultured/environmental sample sequences” from the comparison and referring mainly to CBS collection strains, preferably to ex-type strains. The obtained sequences were deposited in GenBank. To define the phylogenetic position of the isolates within the families Herpotrichiellaceae, Cyphellophoraceae, Trichomeriaceae (order Chaetothyriales), Mycosphaerellaceae (order Mycosphaerellales), and Sympoventuriaceae (order Venturiales), four ITS datasets were generated. Sequences were aligned iteratively with MUSCLE in MEGA6 [40], and the final alignments were improved manually. Maximum likelihood (ML) trees were generated using the Tamura-Nei model, and the robustness of the ML phylogenetic inference was estimated using the bootstrap method [41], with 1000 pseudoreplicates. *Cyphellophora*, *Cladophialophora*, *Cladosporium*, and *Verruconis* were used as outgroups for the Herpotrichiellaceae, Trichomeriaceae, Mycosphaerellaceae, and Sympoventuriaceae trees, respectively.

### 2.4. Black Fungal Diversity

The re-isolation of the same species can occur when samples are processed using different isolation protocols, and/or when multiple samples are collected from the same diesel car tank. Therefore, to assess the black fungal populations in the three studied sites (namely DCT, DPD, and GPD), the general isolation results were analyzed as species presence–absence for each sampling site regardless of the isolation method used or the surface sampled (i.e., inner, external or lid sample as in DCT). A similar presence–absence evaluation scheme was performed to evaluate the different isolation methods. The species isolation frequencies were used to define the black fungal populations in the three studied sites and to calculate the Shannon and Simpson biodiversity indices and the Chao-1 species richness.

### 2.5. Statistical Analysis

Confidence intervals (95% CIs) calculated with the Wald method modified by Agresti–Coull [42] and Chi-square Fisher exact test were used to statistically evaluate differences in isolation frequencies (e.g., by site: DCT, GPD, DPD). PAST software (PAleontological STatistics, ver. 4.06b, [43]) was used to calculate the biodiversity indices and to perform a hierarchical clustering analysis of the black fungal populations using the UPGMA algorithm and the Bray–Curtis similarity index with 100,000 bootstrap replicates.

## 3. Results

### 3.1. General Isolation and Molecular Identification Results

**Figure 2:**
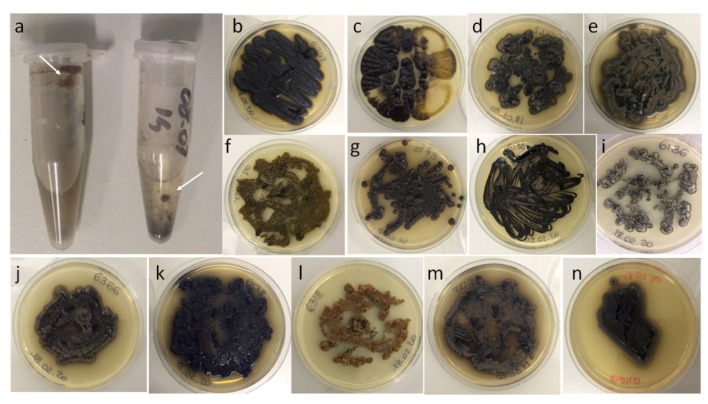
(**a**) Example of sample suspension vials after a long cold incubation (white arrows indicate fungal growth) and pure cultures of selected isolates: (**b**) *E. phaeomuriformis* CCFEE 6242; (**c**) *Aureobasidium melanogenum* CCFEE6236; (**d**) *E. xenobiotica* CCFEE 6142; (**e**) *Exophiala* sp. 2 CCFEE 6334; (**f**) *Aulographina pinorum* CCFEE 6222; (**g**) *E. xenobiotica* CCFEE 6143; (**h**) *E. crusticola* CCFEE 6188; (**i**) *Neodevriesia* sp. CCFEE 6136; (**j**) *K. epidermidis* CCFEE 6366; (**k**) *K. epidermidis* CCFEE 6138; (**l**) *Scolecobasidium* sp. 1 CCFEE 6318; (**m**) *Exophiala* sp. 1 CCFEE 6135; (**n**) *E. heteromorpha* CCFEE 6240.

**Figure 3:**
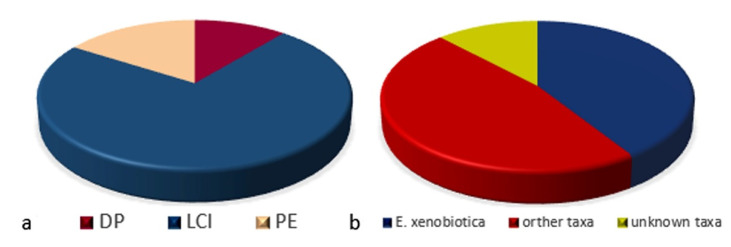
General results based on the 181 isolated strains: (**a**) isolation yields by method: LCI: long cold incubation, DP: direct plating, and PE: phenolic enrichment; (**b**) prevalence of *E. xenobiotica* compared to other known and unknown taxa.

One hundred and eighty-one black fungal isolates were obtained (Figure 2, Figure S1). No significant differences on sample positivity were recorded in the three studied sites (Figure S2a). A total of 87 black fungi were isolated from DCT, 37 from DPD, and 59 from GPD (Figure S2b). Significant differences in isolation yields were recorded when LCI was compared to DP and PE (*p* < 0.00001). The total number of isolates obtained using LCI was up to 6.6 times greater than DP, and 4.6 times greater than PE (Figure 3a). No significant differences were recorded comparing DP and PE results (*p* = 0.3221). Similarly, LCI sample positivity (41.6%, 84/202) was statistically significant (*p* < 0.00001) when compared to PE (10.4%, 21/202) and DP (8.9%, 18/202).

Most of the 181 black fungal isolates were identified at the species level (Table 1). *Exophiala xenobiotica* was the most frequent isolated species (Figure 3b), while 12% of the isolates represented novel fungal taxa. The isolation frequencies in the three niches under investigation were used for the subsequent biodiversity analysis.

### 3.2. Black Fungal Diversity

#### 3.2.1. Black Fungal Diversity in the Three Fuel-Contaminated Sites

Diesel car tanks revealed the highest species richness (21); the difference between the two fuel pump dispensers was slight, being characterized by 13 (DPD) and 14 (GPD) taxa (Table 2).

**Figure 4:**
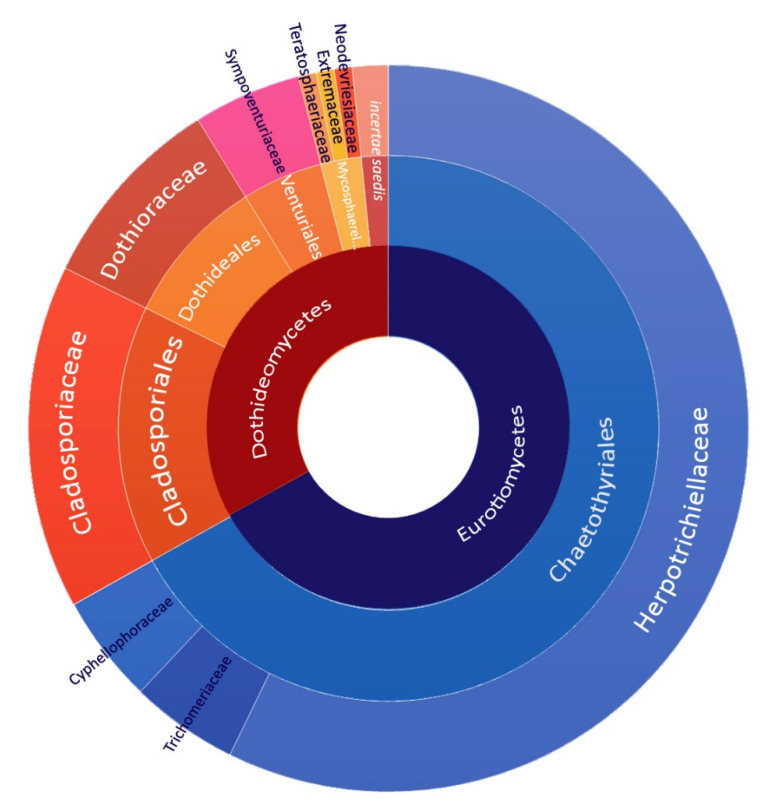
High taxonomic rank of 124 black fungi isolated from DCT (52), DPD (32) and GPD (40). Family (outer ring), order (central ring), and class (internal ring). Different shades of the same color in the chart indicate different families within each order and class.

The order Eurotiomycetes, class Chaetothyriales, and family Herpotrichielaceae were the dominant taxa (Figure 4). The ratio between the classes Eurotiomycetes and Dothideomycetes was about 2:1 (67% vs. 33%). At the order level, Chaetothyriales dominated (67%), followed by Cladosporiales (15%) and Dothideales (8.7%). More than half of all isolates belonged to the family Herpotrichiellaceae (57%), followed by Cladosporiaceae (15%), and Dothioraceae (8.7%). Trichomeriaceae, Cyphellophoraceae (Eurotiomycetes), and Sympoventuariaceae (Dothideomycetes) accounted for less than 5% of the isolates each.

**Figure 5:**
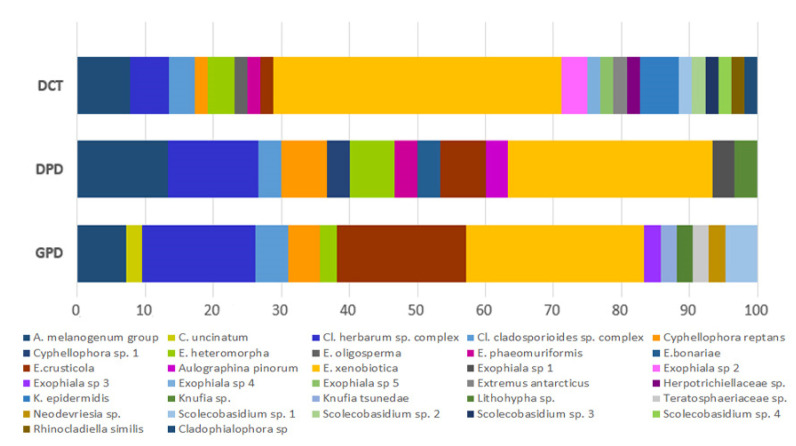
Relative abundance of the cultivable black fungal diversity recorded in diesel car tanks (DCT), diesel pump dispensers (DPD), and gasoline pump dispensers (GPD). The relative abundance is expressed as percentage (x axis).

The diversity at species level is shown in Figure 5. A total of 32 taxa were assigned. In all investigated sites, two groups of cladosporiacean fungi (namely *Cl. herbarum* and *Cl*. *cladosporioides* groups) were found, as well as fungi very close to the type strain of *Aureobasidium melanogenonum*, from 100% to 98.2% in sequence similarity. A more precise species definition within these groups was not possible by using only the ITS sequences. Additionally, *E. crusticola* and *E. xenobiotica* were present in all sites, but their ratio among sites changed from 8:11 in gasoline pump dispensers to 1:22 in diesel car tanks.

**Figure 6:**
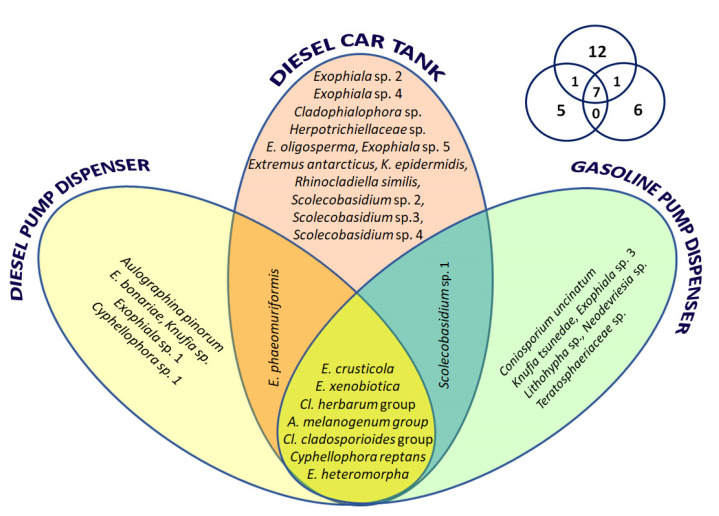
Venn diagram showing the presence of culturable black fungal species detected in diesel car tanks (DCT), diesel pump dispensers (DPD), and gasoline pump dispensers (GPD). On the upper right side, the Venn diagram of species richness is shown.

Of the 32 taxa found, seven taxa were common to all sampling sites, while some were exclusive of one site (Figure 5 and Figure 6): 12 for DCT, five for DPD, and six for GPD. Figure 6 shows the distribution of the new taxa within the studied sites. In particular, three were isolated from DPD (3 out 30 isolates counted, 10%), eight from DCT (9/52, 17%), and five from GPD (5/42, 11%). Hierarchical clustering analysis demonstrated, instead, that populations found on both fuel pump dispensers (namely from GPD and DPD) were more similar than those from DCT (Figure S7).

#### 3.2.2. Black Fungal Diversity with Regard to the Isolation Methods

**Figure 7:**
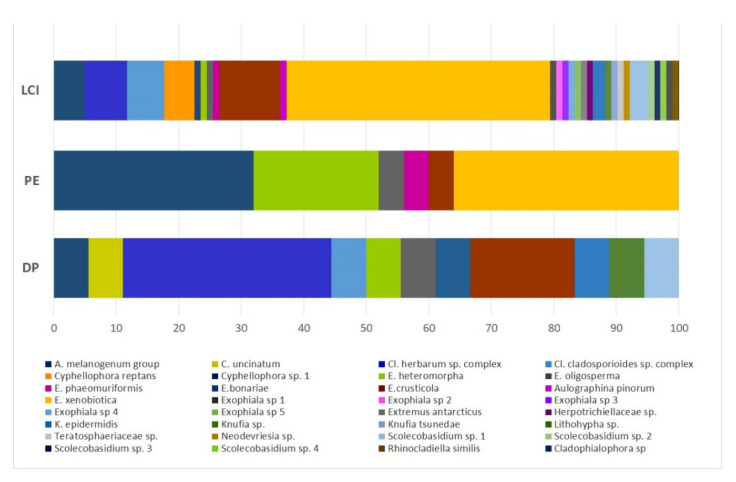
Relative species frequency recorded using the following protocols: long cold incubation (LCI), phenolic enrichment (PE), and direct plating (DP). The species frequency is expressed as percentage (x axis).

Filtered raw data (counting a single species per site and each isolation method) were used to assess the influence of the protocol used on the isolated species. A total of 145 strains were counted, 102 of which were obtained using LCI. The remaining isolates were isolated using PE (25) and DP (18). As shown in Figure 7, about 91% of taxa recorded in this study (29/32) were isolated using LCI. Regarding the 16 new taxa, they were all isolated by LCI (17 strains); while only one was isolated by DP (2 strains). The twenty-five isolates obtained using PE fall into six different taxa*. Exophiala xenobiotica* (36%, yellow), *A. melanogenum (*32%, blue), and *E. heteromorpha* (20%, green) were the most frequent species; no cladosporian species were recorded. The strains isolated by DP fall into 11 taxa, which highlights the absence of *E. xenobiotica* and the prevalence of the ubiquitous *Cladosporium* species complex representing 38.9% (7/18, cobalt blue and light blue) of isolates. Significant differences in PE isolation frequencies were found for *A. melanogenum* (*p* = 0.0005) and *E. heteromorpha* (*p* = 0.001) when compared to LCI, and for *Cladosporium* spp. when the DP results were compared to the other methods (DP vs. LCI *p* = 0113; DP vs. PE *p* = 0.001). When compared to DP, LCI and PE significantly increased the *E. xenobiotica* isolation frequencies (*p* = 0003 and *p* = 0.0056, respectively).

#### 3.2.3. Diversity of New Fungal Taxa

Novel strains accounted for 12% of total isolates (22/181) and 15.3% (19/124) if the general data are filtered to exclude duplicated strains. These unidentified strains belong to 16 different taxa, each generally represented by a single strain. Their higher taxonomic rank distribution (Figure S8) evidenced the Eurotiomycetes prevalence (57.9%). The most frequent families were Herpotrichiellaceae (42.1%) and Sympoventuriaceae (31.6%); the latter was represented exclusively by the *Scolecobasidium* species.

Herpotrichiellaceae sp. CCFEE 6392 possibly belong to a new *Minimelanolacus* species due its position in the ITS tree (Figure S3), and the 98.19% LSU sequences identity with *Minimelanolacus asiaticus* MFLUCC 15-0237 and *Minimelanolacus curvatus* MFLUCC 15-0259. *Cyphellophora* sp. CCFEE 6028 recorded an ITS best match with *Cyphellophora reptans* CBS 113.85 (96.43%) and with *Cyphellophora europaea* CBS 129.96 when LSU was used for comparison (98.76%). Sequencing blasting identified *Cladophialophora chaetospira* as the closest known species to *Cladophialophora* sp. CCFEE 6390 and LSU confirmed this position. Otherwise, Teratosphaeriaceae sp. CCFEE 6137 showed very similar ITS identities, 97.53% and 97.97%, with the ex-type strains of *Mycocalicium victoriae* (Eurotiomycetes) and *Constantinomyces macerans* (Dothideomycetes) respectively, and 96.25% (coverage 99%) with *Patellariopsis dennisii* (Leotiomycetes). However, LSU sequence comparison evidenced *Catenulostroma elginense* CBS 111030 (Dothideomycetes) as the best match (99.34%). No *Mycocalicium victoriae* LSU sequences are available in GenBank, but neither Eurotiomycetes nor Leotiomycetes strains were recorded in the LSU top 100 identity records. The new *Scolecobasidium* species fall into three groups, their closest species are *S. robustum* (*Scolecobasidium* sp. CCFEE 6152, CCFEE 6153, CCFEE 6154), *S. constrictum* (*Scolecobasidium* sp. CCFEE 6388), and *S. globale*, respectively. Two different taxa belonged to this last group, CCFEE 6391 and CCFEE 6363, characterized by different identity scores (Figure S6).

## 4. Discussion

The high-throughput sequencing of environmental DNA has become the dominant method to study microbial diversity in a given habitat. The behavior of individual species can, in fact, be frequently explained in a community context by its interaction with other organisms, which are often unculturable under laboratory conditions [44]. Only a small fraction of the world’s microbial diversity can be cultured under standard microbiological tools and media [45]. If, for bacteria, the “1% culturability paradigm” (recently revised by Martiny [46,47]) is generally accepted (according to which around 99% of them are unculturable), for fungi, it is somehow different. Magnuson and Lasure suggested that 70–90% of soil fungi cannot be obtained by culturing methods [48], but this number is far from being fixed due to the parameters used for evaluation, the habitat considered, and the estimated number of the world’s fungal species [49,50,51,52,53,54]). Whatever this fraction actually is, living pure cultured microorganisms are still necessary for several omics technologies and biotechnological processes and represent the foundation of many in silico applications and for barcoded identifications [55,56].

Three different protocols were used to assess the most suitable method and, additionally, to increase the chances of isolation. Direct plating on DRBC agar (a medium used to inhibit bacteria and slow fast-growing mold) gave poor results due to its insufficient inhibitory power in controlling the spreading of yeasts and sporulating fungi on agar plates at the tested temperature. The PE, already used to isolate black fungi from gasoline car tanks [24], did not give the expected results. The suitability of PE in gasoline car tanks was likely facilitated by the confined conditions and the high accumulation of alkylbenzenes, which are predominant in gasoline and characterize this environment. Despite their resistance to abiotic stresses, black fungi are believed to be potentially vulnerable because of their poor competitive abilities. They indeed may easily be hidden/overwhelmed when conditions become more permissive due to the introduction of a more competitive species [57]. Diesel fuels produce less stringent conditions due to the lower content in volatile aromatics (prevalence of polycyclic aromatic hydrocarbon, PAH) and a variable percentage of hydrotreated vegetal oils (HVO) [58,59]. Fuel dispensers, unlike car fuel tanks, are exposed to the open air, a condition favoring the evaporation of the volatile fraction along with a continuous load with high sporulating fast-growing airborne fungi. The significantly higher isolation yields using LCI (*p* < 0.0001) could be due to a favoring effect on oligotrophic slow-growing organisms able to cope with, and even use, aromatics at low temperatures. Some black fungi such as *E. xenobiotica* MA2853 and *Knufia perforans* MA1299—both chaetothyrialean fungi belonging to Herpotrichiellaceae and Trichomeriaceae families, respectively—are still metabolically active at 1 °C with no significant variations in the number of expressed proteins when compared to optimal temperature conditions [60].

The diversity of black fungi at higher taxonomic ranks is dominated by members of the Eurotiomycetes (class), Chaetothyriales (order), and Herpotrichielaceae (family), similar to other hydrocarbon-contaminated sites [32,61,62]. Concerning the species found (32), the majority were recorded in DCT (21), followed by GPD (14) and DPD (13). Half of the taxa found (50%, 16/32) could not be assigned to known species, resulting occasionally in low identity scores even when a more conserved target is used. These findings highlight how our knowledge of black fungal diversity is still incomplete and largely fragmentary. Interestingly, all 16 unknown taxa were isolated using the LCI protocol, underlining the suitability of this new method in covering the current biodiversity knowledge gap. Unlike standard protocols that are generally rich in carbon sources, LCI maintains oligotrophic conditions; the water availability allows metabolism, and the xenobiotics already present in the sample could work both as a limiting factor and as a carbon source. Moreover, low temperatures slow down the fast-growing cosmopolitan fungi, and the long-period incubation allows black fungi to thrive, thus broadening the isolation chances (Figure S2). The PE might indeed be more selective towards the subset of black fungal strains that most effectively assimilate phenolic compounds, similar to the enrichment of solid state-like fungal cultures on toluene [63].

The species distribution within the three investigated sites evidenced the predominance of well-known species, but also the presence of a number of relatively rare and sometimes unknown species. Seven taxa were common to all sites and included the cosmopolitan genera *Cladosporium*, represented by the *herbarum* and *cladosporioides* groups, and *Aureobasidium*. Isolates belonging to *Cl. herbarum* and *Cl. cladosporioides* species complexes are among the most frequently isolated fungi from the air, being common saprotrophs occurring in an extremely wide range of natural and man-made substrates [64]. The ubiquitous distribution and their growth rate can explain the significant prevalence (*p* < 0.05) of these cosmopolitan fungi when DP (a low-selective method) was used.

As for *Aureobasidium*, all isolated strains shared high sequence identities (up to 100%) with *Aureobasidium melanogenum,* a species previously isolated from oil finished wood and also reported as an occasional pathogen [65,66]. Interestingly, a comparative genomic study on this species has pointed out the possible existence of genes associated with the degradation of plastic and aromatic compounds [67]. This aromatic-degrading potential could explain its significantly higher isolation rate when PE was used as compared to LCI (*p* = 0.0001). Besides their phylogenetic affiliation to the Dothydeomycetes, both *Cladosporium* and *Aureobasidium* species are characterized by an ability to grow under osmophilic conditions [64] and by a relatively hydrophobic cell wall that promotes the adhesion to hydrophobic surfaces [68,69]. These traits may favor the fungal colonization of oily fuel surfaces having a low affinity for water. *Cyphellophora reptans* had previously been isolated from food, human nails and skin scrapings, bark, soil, water [70], and now from fuel-contaminated sites. Three known *Exophiala* species completed the core of fungi found in all the studied sites. *Exophiala xenobiotica,* which is already known for its affinity towards hydrocarbon-rich environments, was the most frequent species representing 40% of total isolates (74/183) recorded in 22 out 25 DCT (88%). The isolation frequencies recorded in the studied sites evidenced that LCI (*p* = 0.0003) and PE (0.0056) can significantly increase the chance of finding *E. xenobiotica* when compared to DP. Additionally, *E. heteromorpha* isolates deserve attention since they were obtained mainly through the PE protocol (5/7, *p* < 0.05), suggesting a possible biodegradative assimilation potential for aromatic compounds. Assimilation of alkylbenzenes has not been demonstrated for *E. heteromorpha* but, just like *E. xenobiotica*, this fungus has been repeatedly isolated from environments rich in hydrocarbons and aromatic compounds [8,37]. The isolation of *E. crusticola*, a soil-associated fungus, was not surprising since it was previously isolated from a creosoted railway tie in cold climates [29,71,72].

Notably, 13 out of the 32 species recorded belong to the polyphyletic genus *Exophiala.* This group is represented by six known species (*E. bonariae, E. crusticola*, *E. heteromorpha, E. oligosperma, E. phaeomuriformis*, and *E. xenobiotica*), five unknown species (CCFEE 6135, 6334, 6348, 6370, and 6387), and two allied taxa (*Rhinocladiella similis* CCFEE 6361, and Herpotrichieallaceae sp. CCFEE 6392) (Table 1). Since 46% of the *Exophiala*-related taxa are unknown, it is reasonable to believe that fuel-contaminated sites could be considered a favorable niche for their finding.

Similarly, *Scolecobasidium*, a genus previously isolated from wet cells and laundry machines [24,73], resulted quite recurrently and is here represented by four different putative new species. There are no previous records on the isolation of *Scolecobasidium* spp. from fuel contaminated sites, and they were mainly isolated using LCI, also suggesting in this case that low temperatures are a factor favoring their occurrence.

The presence of *Coniosporium uncinatum* (CCFEE 6149) and *Knufia epidermidis* (CCFEE 6138, 6198, and 6366) in our study was quite rare, but confirmed previous observations on gasoline car tanks [23]. *Knufia tsunedae* (CCFEE 6411), described as a soil-associated fungus [74], is recorded here for the first time ever in Italy.

In light of the distinct chemical nature of fuels, diesel with predominantly long-chain alkanes and gasoline rich in BTEX, it would be expected that the type of fuel exerts selection pressure on the species characterizing the different populations. Different factors may have concurred regarding the number of isolates and species results, such as the isolation method, the number of samples taken, the geographic area, and the composition of fuels, which can also vary significantly with the brand considered and also with local regulations. Moreover, the environmental influence on the composition of airborne particles cannot be disregarded, as suggested by the finding of plant-associated species such as *Aulographina pinorum* [75] and rock-associated species such as *Extremus antarcticus*, the latter of which was found for the first time in temperate climates [76,77]. Consequently, more samples coming from different countries would be beneficial for gaining a broader knowledge on these contaminated sites in terms of the ecological traits of species, but also from a phylogenetic and taxonomical standpoint. Several authors have demonstrated that Chaetothyriales taxonomy is likely incomplete and deserves a detailed revision [78], but one of the major problems in reconstructing this puzzle is that a huge number of its pieces are still missing. In this context, the hydrocarbon-contaminated niches studied here represent a reservoir for unknown species, which might be brought to light using increasingly specific isolation methods.

Further studies are also necessary to assess the possible dual behavior reported in chaetothyrialean fungi. In this study, we found some species for which a hydrocarbonoclastic potential had already been reported, such as *E. xenobiotica* and *E. oligosperma*, and others whose abilities deserve to be assessed, such as for *E. heteromorpha,* an epiphytic species frequently isolated after phenolic enrichment. Moreover, *E. bonariae* (CCFEE 6041) deserves attention because its type of strain, which was isolated from a marble sculpture, was able to cope with toluene and to grow even at 35 °C (the highest temperature tested) [24,79,80]. Several members of the genus *Exophiala* are potential agents of human and animal mycoses and for that are considered to be in the Bio Safety Level- 2 (BSL-2) group. In this study, together with *E. xenobiotica*, we found other species associated with clinical cases, including *E. oligosperma* a sporadic agent of phaeohyphomycosis [81,82], *E. phaeomuriformis* mentioned in relation to keratitis [83], *Rhinocladiella similis* recently found in a nosocomial infection [84], and *Knufia epidermidis* associated with mild skin infections [85].

Safety issues should also be considered in light of the frequency with which some species have been found. Above all, the case of *E. xenobiotica,* reported as the second most frequent *Exophiala* species in U.S. and Brazilian clinical samples [86,87], is generally associated with opportunistic cutaneous phaeohyphomycoses of a mostly traumatic nature judging from the frequent occurrence of eye, wound, and (sub)cutaneous lesions, mainly in patients with immunodepression or major debilitating diseases [37]. In this study, *E. xenobiotica* was recorded in 73% (22/30) of the sampled cars, and so it is not a surprise if it has been reported recently (maybe by chance) as responsible for cutaneous infections in a car mechanics [88]. The profuse isolation of *Exophiala dermatitidis* (a species associated with deep mycoses in immunocompromised and healthy individuals) [89,90] in a car garage [32] highlights the potential risks for automotive engine and fuel manipulation. Further studies focused on the biodiversity of fungi that colonize devices subjected to both car fuel exposure and human manipulation are essential for a better understanding of pathogenicity and biohazard assessment, and also for their potential use as bioremediation agents.

## 5. Conclusions

The long cold incubation proved to be a powerful tool with which to explore the black fungal diversity in fuel-contaminated sites. Aside from the frequent isolation of *E. xenobiotica*, the finding of several novel taxa and species known for their pathogenic and biodegradative potential further increased interest in hydrocarbon-rich sites. Investigations on fuel-contaminated niches are indeed crucial for defining taxonomic boundaries within Chaethothyriales/Herpotrichiellaceae and assessing the evolutionary route leading to pathogenicity. This research is also essential in evaluating the associated biological risks and selecting species that exhibit a biotechnological potential associated with a low biohazard.

## Figures and Tables

**Table 1:** List of the 181 isolated strains. The identifications were performed by BLASTn comparison and ML ITS-based trees (Figures S3–S6). The taxonomic novelties found were identified at the genus or family level.

Species	CCFEE Collection No	Origin	GenBank Accession No	
			ITS	LSU
*Aulographina pinorum*	6220, 6222, **6230**	DPD, Italy	MZ573423	
*Aureobasidium melanogenum* group	**6141**, 6145, 6146, 6226, 6234, 6235, 6213, 6216, **6227**, 6236, 6407, 6244, 6245, 6246, **6403**, 6408	DCT, DPD, GPD, Italy	MZ573420, MZ573421, MZ573422	
*Cladophialophora* sp.	6390	DCT, Italy	MZ573430	MZ956973
*Cladosporium herbarum* group	6184, **6199**, 6016, 6192, **6193**, **6027**, 6029, 6030, 6031, 6054, 6148, 6191, 6378	DCT, DPD, GPD, Italy	MZ573425, MZ573426, MZ573427	
*Cladosporium cladosporioides* group	**6197**, 6225, **6018**, 6231, 6183, 6218, 6412	DCT, DPD, GPD, Italy	MZ573428, MZ573429	
*Coniosporium* cft. *uncinatum*	**6149**	GPD, Italy	MZ573424	
*Cyphellophora reptans*	**6373**, 6007, 6413, 6379, **6398**	DCT, DPD, GPD, Italy	MZ573432, MZ573434, MZ573433	
*Cyphellophora* sp.	**6028**	DPD, Italy	MZ573431	MZ956974
*Exophiala bonariae*	**6041**	DPD, Italy	MZ573435	
*Exophiala crusticola*	**6039**, 6051, **6058**, 6004, 6005, 6006, 6010, **6014**, 6015, 6019, 6020, 6022, 6023, 6024, 6025, 6026, 6178, 6188, 6224, 6232	DCT, DPD, GPD, Italy	MZ573436, MZ573437, MZ573438	
*Exophiala heteromorpha*	6150, 6181, 6339, 6343, **6240**, 6241, **6243**	DCT, DPD, GPD, Italy	MZ573439, MZ573440	
*Exophiala oligosperma*	**6139**, 6345, 6360	DCT, Italy	MZ573441	
*Exophiala xenobiotica*	**6140**, 6142, 6143, 6179, 6180, 6185, 6186, 6187, 6190, 6196, 6219, 6223, 6229, 6333, 6335, 6340, 6341, 6342, 6344, 6346, 6351, 6352, 6353, 6362, 6364, 6365, 6367, 6368, 6369, 6371, 6372, 6374, 6375, 6376, 6381, 6382, 6383, 6384, 6385, 6386, 6389, 6393, 6394, 6395, 6396, 6397, 6399, 6404, 6157, 6189, 6194, 6195, 6221, 6233, **6237**, 6336, 6337, 6338, 6377, 6410, 6008, **6182**, 6217, 6228, 6238, 6239, 6350, 6354, 6355, 6356, 6357, 6400, 6405, 6406	DCT, DPD, GPD, Italy	MZ573442, MZ573443, MZ573444	
*Exophiala phaeomuriformis*	**6242**, **6358**, 6359	DCT, DPD, Italy	MZ573445, MZ573446	
*Exophiala* sp. 1	**6135**	DPD, Italy	MZ573447	OK178849
*Exophiala* sp. 2	**6334**, 6402	DCT, Italy	MZ573448	MZ956975
*Exophiala* sp. 3	**6348**	GPD, Italy	MZ573449	MZ956976
*Exophiala* sp. 4	**6370**	DCT, Italy	MZ573450	MZ956977
*Exophiala* sp. 5	**6387**	DCT, Italy	MZ573451	MZ956978
*Extremus* cft *antarcticus*	6349	DCT, Italy	MZ573453	
Herpotrichiellaceae sp.	**6392**	DCT, Italy	MZ573459	MZ956980
*Knufia epidermidis*	**6138**, 6198, 6366	DCT, Italy	MZ573455	
*Knufia tsunedae*	**6411**	GPD, Italy	MZ573456	
*Knufia* sp.	**6034**	DPD, Italy	MZ573457	MZ956979
*Lithohypha* sp.	**6069**	GPD, Italy	MZ573458	OK178850
*Neodevriesia* sp.	**6136**	GPD, Italy	MZ573461	OK178852
*Rhinocladiella similis*	6361	DCT, Italy	MZ573467	
*Scolecobasidium* sp. 1	**6154**, 6155, 6156, **6152**, 6153, 6318	DCT, GPD, Italy	MZ573462, MZ573463	MZ956981
*Scolecobasidium* sp. 2	**6363**	DCT, Italy	MZ573464	MZ956982
*Scolecobasidium* sp. 3	**6391**	DCT, Italy	MZ573465	MZ956983
*Scolecobasidium* sp. 4	**6388**	DCT, Italy	MZ573466	MZ956984
Teratosphaeriaceae sp.	**6137**	GPD, Italy	MZ573460	OK178851

Black CCFEE numbers represent the strains isolated from diesel car tanks (DCT); red, those from diesel pump dispensers (DPD); and green, from gasoline pump dispensers (GPD). Bold character indicates strains for which sequences have been deposited in GenBank.

**Table 2:** Isolate data and diversity indices summary for the three investigated sites: diesel car tank (DCT), diesel pump dispensers (DPD), and gasoline pump dispensers (GPD).

	DCT	DPD	GPD	Total
No. of isolates	52	30	42	124
No. of positive sampling sites	25	21	29	75
No. of taxa	21	13	14	32
Shannon index	2.522	2.434	2.367	2.687
Simpson index	0.8145	0.8828	0.8722	0.8558
Chao-1 index	43.31	18.08	19.13	101.4

## Data Availability

Not applicable.

## Supplementary Materials

The following are available online at https://www.mdpi.com/article/10.3390/jof7100817/s1, Table S1. List of the species used to infer the phylogenetic position of the strains in study by ML ITS trees, reported respectively in Figure S3–S6. T indicates ex-type strains. Figure S1. Example of DRBC isolation plates after long cold incubation. Figure S2. Black fungi: general isolation results. (a) Percentage of positive samples (green) by site: DCT: diesel car tank, DPD: diesel pump dispenser, GPD: gasoline pump dispenser; (b) number of isolates by site; for DCT, the different surfaces sampled were reported as IN: internal (red), EXT: external (blue), and: lid (LID, gray). Figure S3 Herpotrichiellaceae ML ITS-based tree generated using the Tamura–Nei model; robustness was estimated using the bootstrap method with 1000 pseudoreplicates; values above 70% are shown. *Cyphellophora* clade was used as outgroup. T indicates type strains; DCT diesel car tanks; DPD diesel pump dispensers; and GPD gasoline pump dispensers. Figure S4. Trichomeriaceae ML ITS-based tree generated using the Tamura–Nei model; robustness was estimated using the bootstrap method with 1000 pseudoreplicates; values above 80% are shown. *Cladophialophora* clade was used as outgroup. T indicates type strains; DCT diesel car tanks; DPD diesel pump dispensers; and GPD gasoline pump dispensers. Figure S5. Mycosphaerellaceae ML ITS-based tree generated using the Tamura–Nei model; robustness was estimated using the bootstrap method with 1000 pseudoreplicates; values above 60% are shown. *Cladosporium* was used as outgroup. T indicates type strains; DCT diesel car tanks; DPD diesel pump dispensers; and GPD gasoline pump dispensers. Figure S6. Sympoventuriaceae ML ITS-based tree generated using the Tamura–Nei model; robustness was estimated using the bootstrap method with 1000 pseudoreplicates. *Verruconis* was used as outgroup. T indicates type strains; DCT diesel car tanks, and GPD gasoline pump dispensers. Figure S7. Hierarchical clustering analysis of the black fungal populations found in diesel car tanks (DCT), diesel pump dispensers (DPD), and gasoline pump dispensers (GPD) using UPGMA algorithm and Bray–Curtis similarity index with 100,000 bootstrap replicates. Cophen. corr. 0.9271. Figure S8. High taxonomic rank of 19, not duplicated, unknown strains. Different shades of the same color in the chart indicate different families within each order and class. The frequency is expressed as percentage (x axis).

## References

[1] Grube M., Muggia L., Gostinčar C., Seckbach J., Oren A., Stan-Lotter H. (2013). Niches and adaptations of polyextremotolerant black fungi. Polyextremophiles.

[2] Selbmann L., Isola D., Egidi E., Zucconi L., Gueidan C., de Hoog G.S., Onofri S. (2014). Mountain tips as reservoirs for new rock-fungal entities: *Saxomyces* gen. nov. and four new species from the Alps. Fungal Divers..

[3] Sterflinger K., Seckbach J. (2005). Black yeasts and meristematic fungi: Ecology, diversity and identification. The Yeast Handbook.

[4] Gandra S., Ram M.S., Levitz S.M. (2021). The “Black Fungus” in India: The emerging syndemic of COVID-19–Associated Mucormycosis. Ann. Intern. Med..

[5] Selbmann L., de Hoog G.S., Zucconi L., Isola D., Onofri S., Buzzini P., Margesin R. (2014). Black yeasts in cold habitats. Cold-adapted Yeasts.

[6] Prenafeta-Boldú F.X., Armjio-Medina C., Isola D., Pacheco-Torgal F., Ivanov V., Falkinham J.O. (2021). Black fungi in the built environment–the good, the bad, and the ugly. Viruses, Bacteria, and Fungi in the Built Environment. Designing Healthy Indoor Environments.

[7] de Hoog G., Vicente V., Najafzadeh M.J., Harrak M., Badali H., Seyedmousavi S. (2011). Waterborne *Exophiala* species causing disease in cold-blooded animals. Persoonia—Mol. Phylogeny Evol. Fungi.

[8] Prenafeta-Boldú F.X., Summerbell R., de Hoog G.S. (2006). Fungi growing on aromatic hydrocarbons: Biotechnology’s unexpected encounter with biohazard?. FEMS Microbiol. Rev..

[9] Sav H., Ozakkas F., Altınbas R., Kiraz N., Tümgör A., Gümral R., Döğen A., Ilkit M., de Hoog G.S. (2016). Virulence markers of opportunistic black yeast in *Exophiala*. Mycoses.

[10] Prenafeta-Boldú F.X., de Hoog G.S., Summerbell R.C., McGenity T.J. (2018). Fungal communities in hydrocarbon degradation. Microbial Communities Utilizing Hydrocarbons and Lipids: Members, Metagenomics and Ecophysiology.

[11] Babič M.N., Zupančič J., Gunde-Cimerman N., de Hoog S., Zalar P. (2018). Ecology of the human opportunistic black yeast *Exophiala dermatitidis* indicates preference for human-made habitats. Mycopathol..

[12] Babič M.N., Zalar P., Ženko B., Džeroski S., Gunde-Cimerman N. (2016). Yeasts and yeast-like fungi in tap water and groundwater, and their transmission to household appliances. Fungal Ecol..

[13] Döğen A., Kaplan E., Öksüz Z., Serin M.S., Ilkit M., de Hoog G.S. (2013). Dishwashers are a major source of human opportunistic yeast-like fungi in indoor environments in Mersin, Turkey. Med. Mycol..

[14] Raghupathi P.K., Zupančič J., Brejnrod A.D., Jacquiod S., Houf K., Burmølle M., Gunde-Cimerman N., Sørensen S.J. (2018). Microbial diversity and putative opportunistic pathogens in dishwasher biofilm communities. Appl. Environ. Microbiol..

[15] Zalar P., Novak M., de Hoog G.S., Gunde-Cimerman N. (2011). Dishwashers—A man-made ecological niche accommodating human opportunistic fungal pathogens. Fungal Biol..

[16] Zupančič J., Babič M.N., Zalar P., Gunde-Cimerman N. (2016). The black yeast *Exophiala dermatitidis* and other selected opportunistic human fungal pathogens spread from dishwashers to kitchens. PLoS ONE.

[17] Hamada N., Abe N. (2009). Physiological characteristics of 13 common fungal species in bathrooms. Mycoscience.

[18] Hamada N., Abe N. (2010). Growth characteristics of four fungal species in bathrooms. Biocontrol Sci..

[19] Lian X., de Hoog G.S. (2010). Indoor wet cells harbour melanized agents of cutaneous infection. Med. Mycol..

[20] Samerpitak K., Gloyna K., Gerrits van den Ende A.H.G., de Hoog G.S. (2017). A novel species of the oligotrophic genus *Ochroconis* colonizing indoor wet cells. Mycoscience.

[21] Wang X., Cai W., Gerrits van den Ende A.H.G., Zhang J., Xie T., Xi L., Li X., Sun J., de Hoog G.S. (2018). Indoor wet cells as a habitat for melanized fungi, opportunistic pathogens on humans and other vertebrates. Sci. Rep..

[22] Hamada N. (2005). Growth on various detergent components of fungi found in washing machines. Seikatsu Eisei.

[23] Hamada N. (2005). Characteristics of fungi growing inside washing machines. Seikatsu Eisei.

[24] Isola D., Selbmann L., de Hoog G.S., Fenice M., Onofri S., Prenafeta-Boldú F.X., Zucconi L. (2013). Isolation and screening of black fungi as degraders of volatile aromatic hydrocarbons. Mycopathologia.

[25] Nucci M., Akiti T., Barreiros G., Silveira F., Revankar S.G., Wickes B., Sutton D.A., Patterson T.F. (2002). Nosocomial outbreak of *Exophiala jeanselmei* fungemia associated with contamination of hospital water. Clin. Infect. Dis..

[26] Nishimura K., Miyaji M., Taguchi H., Tanaka R. (1987). Fungi in bathwater and sludge of bathroom drainpipes. Mycopathologia.

[27] Matos T., de Hoog G.S., de Boer A.G., de Crom I., Haase G.M. (2002). High prevalence of the neurotrope *Exophiala dermatitidis* and related oligotrophic black yeasts in sauna facilities. Mycoses.

[28] Döğen A., Ilkit M., de Hoog G.S. (2013). Black yeast habitat choices and species spectrum on high altitude creosote-treated railway ties. Fungal Biol..

[29] Döğen A., Kaplan E., Ilkit M., de Hoog G.S. (2012). Massive contamination of *Exophiala dermatitidis* and *E. phaeomuriformis* in railway stations in subtropical Turkey. Mycopathologia.

[30] Satow M., Attili-Angelis D., de Hoog G., Vicente V. (2008). Selective factors involved in oil flotation isolation of black yeasts from the environment. Stud. Mycol..

[31] Vicente V., Attili-Angelis D., Pie M., Queiroz-Telles F., Cruz L., Najafzadeh M.J., de Hoog G., Zhao J., Pizzirani-Kleiner A. (2008). Environmental isolation of black yeast-like fungi involved in human infection. Stud. Mycol..

[32] Baron N., Pagnocca F., Otsuka A., Prenafeta-Boldú F., Vicente V., de Angelis D.A. (2021). Black Fungi and Hydrocarbons: An Environmental Survey for Alkylbenzene Assimilation. Microorganisms.

[33] Sudhadham M., Prakitsin S., Sivichai S., Chaiyarat R., Dorrestein G., Menken S., de Hoog G.S. (2008). The neurotropic black yeast *Exophiala dermatitidis* has a possible origin in the tropical rain forest. Stud. Mycol..

[34] Prenafeta-Boldú F.X., Kuhn A., Luykx D.M.A.M., Heidrun A., van Groenestijn J.W., de Bont J.A.M. (2001). Isolation and characterisation of fungi growing on volatile aromatic hydrocarbons as their sole carbon and energy source. Mycol. Res..

[35] Quan Y., Gerrits van den Ende A.H.G., Shi D., Prenafeta-Boldú F.X., Liu Z., Al-Hatmi A.M.S., Ahmed S.A., Verweij P., Kang Y., de Hoog G.S. (2019). A Comparison of isolation methods for black fungi degrading aromatic toxins. Mycopathologia.

[36] Zhao J., Zeng J., de Hoog G.S., Attili-Angelis D., Prenafeta-Boldú F.X. (2010). Isolation and identification of black yeasts by enrichment on atmospheres of monoaromatic hydrocarbons. Microb. Ecol..

[37] de Hoog G.S., Zeng J.S., Harrak M.J., Sutton D.A. (2006). *Exophiala xenobiotica* sp. nov., an opportunistic black yeast inhabiting environments rich in hydrocarbons. Antonie van Leeuwenhoek.

[38] Isola D., Selbmann L., Egidi E., Onofri S., Zucconi L. Black fungi from contaminated sites: One year later. Proceedings of the fifth meeting of the ISHAM working groups on black yeasts and chromoblastomycosis.

[39] Isola D., Zucconi L., Cecchini A., Caneva G. (2021). Dark-pigmented biodeteriogenic fungi in Etruscan hypogeal tombs: New data on their culture-dependent diversity, favouring conditions, and resistance to biocidal treatments. Fungal Biol..

[40] Tamura K., Stecher G., Peterson D., Filipski A., Kumar S. (2013). MEGA6: Molecular Evolutionary Genetics Analysis Version 6.0. Mol. Biol. Evol..

[41] Felsenstein J. (1985). Confidence limits on phylogenies: An approach using the bootstrap. Evolution.

[42] Agresti A., Coull B.A. (1998). Approximate is better than "exact" for interval estimation of binomial proportions. Am. Stat..

[43] Hammer Ø., Harper D.A., Ryan P.D. (2001). Past: Paleontological statistics software package for education and data analysis. Palaeontol. Electron.

[44] Cheeptham N., Cheeptham N. (2013). Advances and challenges in studying cave microbial diversity. Cave Microbiomes: A Novel Resource for Drug Discovery.

[45] Molina-Menor E., Gimeno-Valero H., Pascual J., Peretó J., Porcar M. (2021). High culturable bacterial diversity from a European desert: The Tabernas desert. Front. Microbiol..

[46] Martiny A.C. (2019). High proportions of bacteria are culturable across major biomes. ISME J..

[47] Martiny A.C. (2020). The ‘1% culturability paradigm’ needs to be carefully defined. ISME J..

[48] Magnuson J.K., Lasure L.L. Fungal diversity in soils as assessed by direct culture and molecular techniques. Proceedings of the 102nd General Meeting of the American Society for Microbiology.

[49] Gams W. (2006). Biodiversity of soil-inhabiting fungi. Biodivers. Conserv..

[50] Hawksworth D.L. (1991). The fungal dimension of biodiversity: Magnitude, significance, and conservation. Mycol. Res..

[51] Hawksworth D.L. (2001). The magnitude of fungal diversity: The 1.5 million species estimate revisited. Mycol. Res..

[52] Blackwell M. (2011). The Fungi: 1, 2, 3 … 5.1 million species?. Am. J. Bot..

[53] Hawksworth D.L., Lücking R. (2017). Fungal diversity revisited: 2.2 to 3.8 million species. Microbiol. Spectr..

[54] Wu B., Hussain M., Zhang W., Stadler M., Liu X., Xiang M. (2019). Current insights into fungal species diversity and perspective on naming the environmental DNA sequences of fungi. Mycology.

[55] Kirchman D.L. (2018). Genomes and meta-omics for microbes. Processes in Microbial Ecology.

[56] Hyde K.D., Xu J., Rapior S., Jeewon R., Lumyong S., Niego A.G.T., Abeywickrama P.D., Aluthmuhandiram J.V.S., Brahamanage R.S., Brooks S. (2019). The amazing potential of fungi: 50 ways we can exploit fungi industrially. Fungal Divers..

[57] Selbmann L., Isola D., Fenice M., Zucconi L., Sterflinger K., Onofri S. (2012). Potential extinction of Antarctic endemic fungal species as a consequence of global warming. Sci. Total. Environ..

[58] Dobrzyńska E., Szewczynska M., Pośniak M., Szczotka A., Puchałka B., Woodburn J. (2019). Exhaust emissions from diesel engines fueled by different blends with the addition of nanomodifiers and hydrotreated vegetable oil HVO. Environ. Pollut..

[59] Directive 2009/30/EC of the European Parliament and of the Council of 23 April 2009 Amending Directive 98/70/EC as Regards the Specification of Petrol, Diesel and Gas-Oil and Introducing a Mechanism to Monitor and Reduce Greenhouse Gas Emissions and Amending Council Directive 1999/32/EC as Regards the Specification of Fuel Used by Inland Waterway Vessels and Repealing Directive 93/12/EEC (Text with EEA relevance)—Document 32009L0030. https://eur-lex.europa.eu/legal-content/EN/TXT/?uri=celex%3A32009L0030.

[60] Tesei D., Marzban G., Zakharova K., Isola D., Selbmann L., Sterflinger K. (2012). Alteration of protein patterns in black rock inhabiting fungi as a response to different temperatures. Fungal Biol..

[61] Schwarz A., Adetutu E.M., Juhasz A.L., Aburto-Medina A., Ball A.S., Shahsavari E. (2018). Response of the fungal community to chronic petrogenic contamination in surface and subsurface soils. Geoderma.

[62] Teixeira M.M., Moreno L.F., Stielow B., Muszewska A., Hainaut M., Gonzaga L., Abouelleil A., Patané J.S.L., Priest M., Souza R. (2017). Exploring the genomic diversity of black yeasts and relatives (Chaetothyriales, Ascomycota). Stud. Mycol..

[63] Nascimento M.M., Vicente V.A., Bittencourt J.V., Gelinski J.M.L., Prenafeta-Boldú F.X., Romero-Güiza M., Fornari G., Gomes R.R., Santos G.D., Gerrits van den Ende A.H.G. (2017). Diversity of opportunistic black fungi on babassu coconut shells, a rich source of esters and hydrocarbons. Fungal Biol..

[64] Domsch K.H., Gams W., Anderson T.H. (1980). Compendium of Soil Fungi.

[65] Chen W.-T., Tu M.-E., Sun P.-L. (2016). Superficial phaeohyphomycosis caused by *Aureobasidium melanogenum* mimicking tinea nigra in an immunocompetent patient and review of published reports. Mycopathologia.

[66] Van Nieuwenhuijzen E.J., Houbraken J.A.M.P., Meijer M., Adan O.C.G., Samson R.A. (2016). *Aureobasidium melanogenum*: A native of dark biofinishes on oil treated wood. Antonie van Leeuwenhoek.

[67] Gostinčar C., Ohm R.A., Kogej T., Sonjak S., Turk M., Zajc J., Zalar P., Grube M., Sun H., Han J. (2014). Genome sequencing of four *Aureobasidium pullulans* varieties: Biotechnological potential, stress tolerance, and description of new species. BMC Genom..

[68] Chau H., Si B.C., Goh Y.K., Vujanovic V. (2009). A novel method for identifying hydrophobicity on fungal surfaces. Mycol. Res..

[69] Webb J., Van der Mei H.C., Nixon M., Eastwood I.M., Greenhalgh M., Read S.J., Robson G.D., Handley P.S. (1999). Plasticizers increase adhesion of the deteriogenic fungus *Aureobasidium pullulans* to polyvinyl chloride. Appl. Environ. Microbiol..

[70] Réblová M., Untereiner W.A., Réblová K. (2013). Novel evolutionary lineages revealed in the Chaetothyriales (Fungi) based on multigene phylogenetic analyses and comparison of ITS secondary structure. PLoS ONE.

[71] Gümral R., Tümgör A., Saraçli M.A., Yildiran S.T., Ilkit M., de Hoog G.S., Saraçlı M.A., Yıldıran Ş.T. (2014). Black yeast diversity on creosoted railway sleepers changes with ambient climatic conditions. Microb. Ecol..

[72] Bates S.T., Reddy G.S.N., Garcia-Pichel F. (2006). *Exophiala crusticola* anam. nov. (affinity Herpotrichiellaceae), a novel black yeast from biological soil crusts in the Western United States. Int. J. Syst. Evol. Microbiol..

[73] Samerpitak K., Duarte A.P.M., Attili-Angelis D., Pagnocca F.C., Heinrichs G., Rijs A.J.M.M., Alfjorden A., Gerrits van den Ende A.H.G., Menken S.B.J., de Hoog G.S. (2015). A new species of the oligotrophic genus *Ochroconis* (Sympoventuriaceae). Mycol. Prog..

[74] Madrid H., Hernández-Restrepo M., Gené J., Cano J., Guarro J., Silva V. (2016). New and interesting chaetothyrialean fungi from Spain. Mycol. Prog..

[75] Cheewangkoon R., Groenewald J.Z., Hyde K.D., To-Anun C., Crous P.W. (2010). Chocolate spot disease of *Eucalyptus*. Mycol. Prog..

[76] Selbmann L., Zucconi L., Isola D., Onofri S. (2014). Rock black fungi: Excellence in the extremes, from the Antarctic to space. Curr. Genet..

[77] Schiaparelli S., Selbmann L., Onofri S., Zucconi L., Isola D., Rottigni M., Ghiglione C., Piazza P., Alvaro M.C. (2015). Distributional records of Antarctic fungi based on strains preserved in the Culture Collection of Fungi from Extreme Environments (CCFEE) Mycological Section associated with the Italian National Antarctic Museum (MNA). MycoKeys.

[78] Quan Y., Muggia L., Moreno L.F., Wang M., Al-Hatmi A.M.S., Menezes N.D.S., Shi D., Deng S., Ahmed S., Hyde K.D. (2020). A re-evaluation of the Chaetothyriales using criteria of comparative biology. Fungal Divers..

[79] Isola D., Zucconi L., Onofri S., Caneva G., de Hoog G.S., Selbmann L. (2016). Extremotolerant rock inhabiting black fungi from Italian monumental sites. Fungal Divers..

[80] Isola D., Selbmann L., Meloni P., Maracci E., Onofri S., Zucconi L., Rogerio-Candelera M.A., Lazzari M., Cano E. (2013). Detrimental rock black fungi and biocides: A study on the monumental cemetery of Cagliari. Science and Technology for the Conservation of Cultural Heritage.

[81] González-López M.A., Salesa R., González-Vela M.C., Fernández-Llaca H., Val-Bernal J.F., Cano-Lira J.F. (2007). Subcutaneous phaeohyphomycosis caused by *Exophiala oligosperma* in a renal transplant recipient. Br. J. Dermatol..

[82] Venkateshwar S., Ambroise M.M., Asir G.J., Mudhigeti N., Ramdas A., Authy K., Shivaprakash M.R., Kanungo R. (2014). A rare case report of subcutaneous phaeohyphomycotic cyst caused by *Exophiala oligosperma* in an immunocompetent host with literature review. Mycopathologia.

[83] Vicente A., Domellöf F.P., Byström B. (2018). *Exophiala phaeomuriformis* keratitis in a subarctic climate region: A case report. Acta Ophthalmol..

[84] Abdolrasouli A., Gibani M.M., de Groot T., Borman A.M., Hoffman P., Azadian B.S., Mughal N., Moore L.S., Johnson E.M., Meis J.F. (2021). A pseudo-outbreak of *Rhinocladiella similis* in a bronchoscopy unit of a tertiary care teaching hospital in London, United Kingdom. Mycoses.

[85] Chowdhary A., Perfect J., de Hoog G.S. (2014). Black molds and melanized yeasts pathogenic to humans. Cold Spring Harb. Perspect. Med..

[86] Zeng J.S., Sutton D.A., Fothergill A.W., Rinaldi M.G., Harrak M.J., de Hoog G.S. (2007). Spectrum of clinically relevant *Exophiala* species in the United States. J. Clin. Microbiol..

[87] Silva W.C., Gonçalves S.S., Santos D.W.C.L., Padovan A.C.B., Bizerra F.C., Melo A.S.A. (2017). Species diversity, antifungal susceptibility and phenotypic and genotypic characterisation of *Exophiala* spp. infecting patients in different medical centres in Brazil. Mycoses.

[88] Espanhol C.M., Recuero J.K., Pagani D.M., Ribeiro A.C., Vettorato G., Duquia R.P., Luzzatto L., Scroferneker M.L. (2019). Cutaneous phaeohyphomycosis caused by *Exophiala xenobiotica*: A case report. Med. Mycol. Case Rep..

[89] Moreno L.F., Vicente V., de Hoog G.S. (2018). Black yeasts in the omics era: Achievements and challenges. Med. Mycol..

[90] Sood S., Vaid V., Sharma M., Bhartiya H. (2014). Cerebral phaeohyphomycosis by *Exophiala dermatitidis*. Indian J. Med. Microbiol..

