# Supplementary material for: Hydrocarbon-Contaminated Sites: Is There Something More Than Exophiala xenobiotica? New Insights into Black Fungal Diversity Using the Long Cold Incubation Method
Source: J Fungi (Basel). 2021 Sep 29;7(10):817. doi: 10.3390/jof7100817 (PMC8538888; doi:10.3390/jof7100817)
Supplement: Supplementary file 1 [file jof-07-00817-s001.zip › jof-1326457/Table S1.pdf]

Table S1. List of the species used to infer the phylogenetic position of the strains in study by ML ITS trees, reported respectively in Figure S3-S6. T indicates ex-type strains.

Species ID	Collection No	Source	Country	Accession No	References
Herpotrichiellaceae ITS tree					
Cyphellophora europaea	CBS 656.82	Human nail	France	JQ766442	[1]
Cyphellophora olivacea	IFM 53357	-	-	AB190379	[1]
Cyphellophora olivacea	CCFEE 6619	Etruscan tomb	Italy	MT472271	[2]
Cyphellophora reptans	CBS 113.85 T	Foodstuff	Sweden	JQ766445	[1]
Cyphellophora sessilis	CBS 243.85 T	Resin of *Picea abies*	Netherlands	EU514700	[3]
Cyphellophora sessilis	CBS 238.93	Styrene air biofilter	Netherlands	AY857541	[3]
Cyphellophora sp.	CCFEE 6668	Etruscan tomb	Italy	MT472288	[1]
Exophiala bergeri	CBS 353.52 T	Chromomycosis, man	Canada	EF551462	[4]
Exophiala bergeri	UTHSC94-540	Index finger	USA	EF025404	[4]
Exophiala bonariae	CBS 139957 T = CCFEE 5792	Marble, funerary monument	Italy	JX681046	[5]
Exophiala bonariae	CCFEE 5899	Marble, funerary monument	Italy	KP791795	[5]
Exophiala capensis	CBS 128771 T = CPC 18473	Leaf bracts of *Phaenocoma prolifera*	South Africa	JF499841	[6]
Exophiala castellanii	CBS 158.58 T = ATCC 18657 = IFM 4702 = MUCL 10097	Skin scrapings, human patient	Sri Lanka	MH857734	[7]
Exophiala castellanii	CBS 110025	Drinking water	Germany	JF747072	[7]
Exophiala crusticola	CP141b T	Soil crust	USA	AM048755	[8]
Exophiala dermatitidis	CBS 207.35 T	Facial chromomycosis	Japan	AF050269	[9]
Exophiala dermatitidis	CBS109154	Brain human	South Korea	AY857525	[9]
Exophiala dermatitidis	NH 166	bathroom	Japan	AB498923	[9]
Exophiala equina	CBS 119.23 T	Horse	Italy	JF747094	[7]
Exophiala equina	IFM 46120	Soil	Brazil	LC600987	[7]
Exophiala heteromorpha	CBS 633.69= DAOM 75853k = MUCL 15475	Railway tie	Canada	AY857522	[9]
Exophiala heteromorpha	CBS 232.33 T== CDC B-2823 = MUCL 9894	Wood pulp	Sweden	AY857524	[9]
Exophiala jeanselmei	CBS 528.76= ATCC 10224	Skin, human	–	AY857530	[10]
Exophiala jeanselmei	CBS 507.90 T	Man, mycethoma	Uruguay	AF050271	[10]
Exophiala lecanii-corni	CBS 123.33	Scale insect, *Lecanium corni*	–	AY857528	[9]
Exophiala lecanii-corni	CBS 102400	Air biofilter	USA	AY857527	[9]
Exophiala lecanii-corni	CCFEE 5689	Soap dispenser washing machine	Italy	JX681038	[11]
Exophiala mesophila	CBS 402.95 T	Shower joint	Germany	JF747111	[7]

Exophiala mesophila	CBS 119910	Dental waterline	USA	JF747113	[7]
Exophiala mesophila	CCFFEE 5821	Kitchen sponge	Italy	JX681041	[11]
Exophiala nigra	CBS 535.94 T	Arctic water	Russia	MH862481	[4]
Exophiala nigra	CBS 546.82	Arctic water	Russia	MH861525	[4]
Exophiala oligosperma	CBS 113408	Toluene air biofilter	Spain	AY857531	[9]
Exophiala oligosperma	CBS 725.88 T	Tumor, human	Germany	AY163551	[9]
Exophiala oligosperma	CBS 124190	Bathroom flask	the Netherlands	GQ426977	[9]
Exophiala oligosperma	CBS 579.76	Brain, human	Japan	AY857533	[9]
Exophiala opportunistica	CBS 109811 T	Drinking water	Germany	JF747123	[9]
Exophiala phaeomuriformis	CBS 124194	Bathroom flask	the Netherlands	GQ426970	[12]
Exophiala phaeomuriformis	CBS 137224	Concrete railway sleeper	Turkey	KJ522802	[12]
Exophiala pisciphila	CBS 537.73 T	Channel catfish, Alabama	USA	AF050272	[7]
Exophiala pisciphila	CCF 4488	Stomach of *Paracheirodon axelrodi*	Czech Republic	LT604105	[7]
Exophiala salmonis	CBS 157.67 T	Cerebral mycetoma	Canada	AF050274	[7]
Exophiala salmonis	CBS 110371	Drinking water	the Netherlands	JF747139	[7]
Exophiala sideris	CBS 121818 T	Berry of *Sorbus aucuparia*	the Netherlands	HQ452311	[13]
Exophiala sideris	CBS 121828	Oak railway tie	the Netherlands	HQ452325	[13]
Exophiala sp.	CCFEE 6622	Etruscan tomb	Italy	MT472280	[1]
Exophiala spinifera	CBS 899.68 T=ATCC 18218 = DSM 1217	Nasal granuloma, human	USA	AY156976	[4]
Exophiala spinifera	CBS 101537	Cactus	Venezuela	AY156970	[4]
Exophiala xenobiotica	CBS 118157 T	Oil contaminated soil	Venezuela	NR_11120	[14]
Exophiala xenobiotica	CBS 117647=UTHSC 00-2163	Wrist wound, human	USA	KP132147.	[14]
Exophiala xenobiotica	CBS 522.76	Decaying timber	UK	KP132164	[14]
Minimelanolcus melanicus	MFLUCC 15-0415 T	Decaying wood submerged in a stream	China	KR215608	[15]
Minimelanolocus curvatus	MFLUCC 15-0259 T	Decaying wood submerged in a stream	China	KR215614	[15]
Rhinocladiella similis	dH 13054	Brain, human	Slovenia	AY857529	[10]
Rhinocladiella similis	CBS 120259	biofilter eliminating gasoline vapors	-	KP132555	[16]
Rhinocladiella similis	CBS 111763 T	Chronic cutaneous ulcer, human	Brazil	MH862885	[10]
Trichomeriaceae ITS tree					
Bradymyces alpinus	CCFEE 5493 T=CBS138368 = CCF 4803	Natural rock, Monte Rosa	Italy	NR_132844	[17]
Bradymyces graniticola	CCF 5193 T	Granite wall of a metro station	Sweden	NR_153653	[18]
Bradymyces oncorhynchi	CCF 4369 T= CBS 133066 = CCFEE 6134	Spleen of *Oncorhynchus mykiss*	Czech Republic	HG426062	[17]

Knufia aspidioti	BJ01A29	Scale insect, *Aspidioti* sp.	China	KF589944	[19]
Knufia aspidioti	BJ01A10	Scale insect, *Aspidioti* sp.	China	KF589943	[19]
Knufia cryptophialidica	DAOM 216555 T	Black galls, *Populus tremuloides*	Canada	JN040501	[20]
Knufia endospora	UAMH 10396	Bark, *Populus tremuloides*	Canada	JN040510	[21]
Knufia epidermidis	CBS 120353	Skin infection	China	EU730589	[22]
Knufia epidermidis	CBS 123466	Nail with onychomycosis	China	FJ355955	[22]
Knufia mediterranea	CCFEE 6205	Marble, funerary monument	Italy	KP791794	[23]
Knufia mediterranea	CCFEE 6211	Marble, funerary monument	Italy	KP791793	[23]
Knufia peltigerae	UAMH 11090	thalli of *Peltigera rufescens*	Luxembourg	HQ709322	[23]
Knufia peltigerae	CGMCC 3.17296	Rock	China	KP226561	[24]
Knufia perfecta	CPC 12173	*Prunus* sp.	Canada	EU035422	[25]
Knufia perfecta.	IRAN 2553C T	Dead branch, *Juglans regia*	Iran	MF062036	[25]
Knufia perforans	CBS 885.95 T	Marble	Greece	AJ244230	[22]
Knufia petricola	CBS 123.872= A95	Marble statue	Italy	KC978734	[23]
Knufia petricola	CBS 726.95 T	Marble statue	Italy	KC978746	[26]
Knufia petricola	CBS 725.95	Calcaremite obelisque	Greece	KC978745	[23]
Knufia petricola	IMI 38917=ch49	Marble	Crimea	AJ507323	[23]
Knufia separata	CGMCC 3.17337	Rock	China	KP174856	[24]
Knufia sp.	CCFEE 5323	Thallus of *Lecanora* sp	Antarctica	FJ392866	[22]
Knufia sp.	CCFEE 5314	Thallus of *Xanthoria elegans*	Antarctica	FJ392865	[22]
Knufia sp.	CCFEE 5817	Gasoline car tank	Italy	Unpublished	[11]
Knufia sp.	MUT4888	Mediterranean Sea, *Flabellia petiolata*	Italy	KR014348	Unpublished
Knufia tsunedae	CPC 22931 T	Soil	Spain	HG003669	[27]
Knufia vaticanii	CBS 139722 T=CCFEE 5939	Travertine	Vatican City State	KP791780	[23]
Lithohypha aloicola	CPC 35996 T	Leaves of *Aloe* sp.	South Africa	MN562103	[28]
Lithohypha guttulata	CCFEE 5884	Marble	Vatican City State	KP791768	[23]
Lithohypha guttulata	CCFEE 5909	Marble	Vatican City State	KP791771	[23]
Metulocladosporiella musicola	CBS113873	*Musa sapientum*	Mozambique	DQ008135	[29]
Metulocladosporiella musicola	CBS 110960 T	*Musa acuminata*	South Africa	DQ008127	[29]
Metulocladosporiella musicola	CBS113864	*Musa* sp.	Uganda	DQ008133	[29]
Metulocladosporiella musicola	CBS113865	*Musa sapientum*	Uganda	DQ008134	[29]
Trichomerium deniqulatum	MFLUCC 10-0884 T	Leaf of *Psidium guajava*	Thailand	JX3136554	[30]
Trichomerium foliicola	MFLUCC 10-0078 T	Leaf of *Murray apaniculata*	Thailand	JX313655	[30]
Trichomerium foliicola	MFLUCC 10-0054	*Mangifera indica*	Thailand	JX313651	[30]
Mycosphaerellaceae ITS tree					
Arthrocatena tenebrio	CBS 136100= CCFEE 5413	Punta Indren, Monte Rosa	Italy	KF309948	[31]

Cladosporium herbarum	CBS 121621=CPC 12177	*Hordeum vulgare*	the Netherlands	MH863124	[32]
Cladosporium hillianum					
Cladosporium iridis	CBS 138.40T = CPC 15458	*Iris* sp.	the Netherlands	EF679370	[33]
Hyphoconis sterilis	CBS 118321= TRN287	Natural rocks, Central Mountain System	Spain	AY843126	[31]
Neodevriesia sp.	CBS 118302 = TRN142	Rock sample: Mallorca	Spain	AY559374	[31]
Neodevriesia agapanthi	CBS 132689 T=CPC 19833	Leaves of *Agapanthus africanus*	South Africa	JX069875	[31]
Neodevriesia bulbillosa	CBS 118285 T=TRN81	Limestone, Mallorca	Spain	AY559341	[31]
Neodevriesia capensis	CBS 130602 T = CPC 18299	*Protea* sp.	South Africa	JN712501	[28]
Neodevriesia capensis	CPC 13981	*Protea repens*	South Africa	EU707887	[28]
Neodevriesia imbrexigena	CAP 1371	Glazed decorative tiles in association with *Trebouxia* sp.	Portugal	JX915745	[34]
Neodevriesia imbrexigena	CAP 1373	Glazed decorative tiles in association with *Trebouxia* sp.	Portugal	JX915746	[34]
Neodevriesia knoxdaviesii	CBS 122898=CPC 14905	*Protea* sp.	South Africa	EU707866	[28]
Neodevriesia lagerstroemiae	CBS 125422 T=CPC 14403	*Lagerstroemia indica*	USA	GU214634	[28]
Neodevriesia modesta	CBS 137182 T=CCFEE 5672	Wallpainting, Cave,	Italy	KF309984	[31]
Neodevriesia queenslandica	CBS 129527 T = CPC 17306	*Scaevola taccada*	Australia	JF951148	[28]
Neodevriesia sardiniae	CBS 139724 T = CCFEE 6202	Marble, funerary monument	Italy	KP791765	[23]
Neodevriesia sardiniae	CCFEE 6210	Marble, funerary monument	Italy	KP791766	[23]
Neodevriesia shakazului	CBS 133579 T= CPC 19784	Leaves of *Aloe* sp.	South Africa	KC005776	[28]
Neodevriesia simplex	CBS 137183 T=CCFEE 5681	Wallpainting, Cave	Italy	KF309985	[31]
Neodevriesia stirlingiae	CBS 133581 T=CPC 19948E	leaves of *Stirlingia latifolia*	Australia	KC005778	[28]
Neodevriesia strelitziae	CBS 122379 T	leaves of *Strelitzia nicolai*	South Africa	EU436763	[28]
Neodevriesia sp.	CBS 118302=TRN142	Limestone, Mallorca	Spain	AY559374	[31]
Extremus sp.	CBS 18300=TRN137	Limestone, Mallorca	Spain	AY559369	[31]
Extremus adstrictus	CBS 118292 T= TRN96	Limestone, Mallorca	Spain	AY559346	[31]
Extremus antarcticus	CBS 136104=CCFEE 5207	Rock, Inexpressible Island	Antarctica	KF309980	[31]
Extremus antarcticus	CCFEE 5312	Rock, Vegetation Island	Antarctica	KF309979	[31]
Petrophila incerta	CBS 118305=TRN62	Limestone, Mallorca	Spain	AY559328	[31]
Petrophila incerta	CBS 118608 T=TRN139b	Limestone, Mallorca	Spain	AY559372	[31]
Saxophila tyrrhenica	CBS 139725 T = CCFEE 5935	Marble angels	Italy	KP791764	[23]
Saxophila tyrrhenica	TRN65	Limestone, Mallorca	Spain	AY559329	[23]
Vermiconidia antarctica	CBS 136107 T=CCFEE 5488	Rock, Battleship Promontory	Antarctica	KF309982	[31]
Vermiconidia antarctica	CBS 136108=CCFEE 5489	Rock, Battleship Promontory	Antarctica	KF309983	[31]
Vermiconia flagrans	CBS 118283 T=TRN124	Limestone, Mallorca	Spain	AY559359	[31]
Vermiconidia flagrans	CBS 118284=TRN104	Limestone, Mallorca	Spain	AY559348	[31]

Sympoventuriaceae ITS tree					
Scolecobasidium anellii	CBS 284.64 T	Cartellana cave, stalactites	Italy	FR832477	[35]
Scolecobasidium anomalum	CBS 131816 T	Lascaux cave, painted gallery	France	HE575201	[35]
Scolecobasidium aquaticum	CBS 140316 T	cold shower of fish-processing company	Germany	NR_164397	[20]
Scolecobasidium bacilliforme	CBS 100442 T	biofilm on stainless steel in drinking water	Germany	NR_155566	[36]
Scolecobasidium capsici	CPC 28782 T	Capsicum annuum	Thailand	NR_155602	[37]
Scolecobasidium constricta	CBS 202.27 T	Soil	USA	AB161063	[37]
Scolecobasidium cordanae	CBS 475.80 T	Dead leaf	Colombia	KF156022	[38]
Scolecobasidium dracaenae	CBS 141323	Leaf spot, *Dracaena* sp.	USA	NR_145404	[39]
Scolecobasidium gamsii	CBS 239.78 T	Plant leaf	Sri Lanka	KF156019	[38]
Scolecobasidium globale	CBS 119644 T	Domestic sample	Germany	KF961086	[40]]
Scolecobasidium humicola	CBS 116655 T	Peat soil	Canada	HQ667521	[41]
Scolecobasidium icarus	CBS 536.69 T	Forest soil	Canada	HQ667524	[38]
Scolecobasidium lascauxense	CBS 131815 T	Lascaux cave, passageway	France	FR832474	[35]
Scolecobasidium longiphorum	CBS 435.76	Soil	Canada	KF156038	[41]
Scolecobasidium macrozamiae	CBS 102491	*Macrozamia* (Zamiaceae) leaf litter	Australia	KF156021	[42]
Scolecobasidium minimum	CBS 510.71 T	Rhizosphere	Nigeria	HQ667522	[41]
Scolecobasidium musae	CBS 729.95	Fruit surface of Japanese fiber banana, *Musa basjoo*	China	KF156029	[20]
Scolecobasidium olivaceum	CBS 137170 T	Bronchoalveolar fluid	USA	LM644521	[38]
Scolecobasidium pandanicolum	CPC 263170 T	Leaves of *Pandanus* sp.	France	NR145399	[43]
Scolecobasidium podocarpi	CPC 37078 T=CBS 143174	*Podocarpus grayae*	Australia	NR_156654	[43]
Scolecobasidium ramosum	CBS 137173 T	Nail	USA	LM644524	[38]
Scolecobasidium robustum	CBS 112.97 T	Leaf litter of *Ouercus ilex*	Spain	NR_155567	[36]
Scolecobasidium sexuale	CBS 131765 T	Domestic sample	South Africa	KF156018	[38]
Scolecobasidium tshawytschae	CBS 100438 T	Fish	USA	HQ667562	[41]
Scolecobasidium verrucosum	CBS 383.81 T	Soil	India	KF156015	[42]
Scolecobasidium ailanthi	MFLUCC 17-0923	*Ailanthus sp.*	Thailand	NR_163326	[44]
Scolecobasidium phaeophorum	CBS 206.96	Leaf coastal rain forest	Papua New Guinea	NR_155565	[36]
Verruconis calidifluminalis	IFM 54738	Hot spring river	Japan	AB385698	[45]
Verruconis gallopava	CBS 437.64	Brain	Turkey	HQ667553	[45]

ATCC American Type Culture Collection, Rockville, Maryland, USA; *CAP* Culture Collection of Alan Phillips, housed at M&B-BioISI, Tec Labs, University of Lisbon, Portugal; *CBS* CBS Fungal Biodiversity Centre, Centraalbureau voor Schimmelcultures, Utrecht, The Netherlands; *CCF* Culture Collection of Fungi, Dept of Botany, Charles University, Prague, Czech Republic; *CCFEE* Culture Collection of Fungi from Extreme Environments, Dept. of Ecological and Biological Sciences, University of Tuscia, Viterbo, Italy; *CDC* Centers for Disease Control and Prevention, Atlanta, Georgia, USA, *CGMCC* China General Microbiological Culture Collection Center Institute of Microbiology ChineseAcademy of Sciences Beijing China; *CPC* Collection Pedro Crous, housedat CBS; *DSMZ* Deutsche Sammlung von Mikroorgannismen und Zellkulturen, Baunschweig, Germany; *DAOM* Plant Research Institute, Department of Agriculture (Mycology), Ottawa, Canada;, dH Culture collection of Sybren de Hoog, housed at

CBS; IFM Research Center for Pathogenic Fungi and Microbial Toxicoses, Chiba, Japan; *IMI* International Mycological Institute Collection is embedded in the CABI UK Centre in Egham, UK; IRAN Iranian fungal culture collection part of the Herbarium Ministerii Iranici Agriculturae; Tehran, Iran MFLUCC: Mae Fah Luang University Culture Collection, Thailand; *MUCL* Mycothèque de l'Université catholique de Louvain, Louvain-la-Neuve, Belgium; *MUT* Mycotheca Universitatis Taurinensis, Dept of life Sciences, University of Torino; *NH* Nabuo Hamada personal collection; *TRN* T. Ruibal personal collection; *UAMH* University of Alberta Microfungus Collection and Herbarium, Canada, *UTHSC* University of Texas Health Science, Center Fungus Testing Laboratory -Dept. of Pathology, San Antonio, Texas, U.S.A.

References

1. Feng, P.; Lu, Q.; Najafzadeh, M.J.; Gerrits van den Ende, A.H.G.; Sun, J.; Li, R.; Xi, L.; Vicente, V.A.; Lai, W.; Lu, C.; et al. *Cyphellophora* and its relatives in *Phialophora*: Biodiversity and possible role in human infection. *Fungal Divers.* **2012**, *65*, 17–45, doi:10.1007/s13225-012-0194-5.
2. Isola, D.; Zucconi, L.; Cecchini, A.; Caneva, G. Dark-pigmented biodeteriogenic fungi in Etruscan hypogeal tombs: New data on their culture-dependent diversity, favouring conditions, and resistance to biocidal treatments. *Fungal Biol.* **2021**, doi:10.1016/j.funbio.2021.03.003.
3. Gao, L.; Ma, Y.; Zhao, W.; Wei, Z.; Gleason, M.L.; Chen, H.; Hao, L.; Sun, G.; Zhang, R. Three new species of *Cyphellophora* (chaetothyriales) associated with sooty blotch and flyspeck. *PLoS ONE* **2015**, *10*, e0136857, doi:10.1371/journal.pone.0136857.
4. Zeng, J.S.; de Hoog, G.S. *Exophiala spinifera* and its allies: Diagnostics from morphology to DNA barcoding. *Med. Mycol.* **2008**, *46*, 193–208, doi:10.1080/13693780701799217.
5. Isola, D.; Zucconi, L.; Onofri, S.; Caneva, G.; de Hoog, G.S.; Selbmann, L. Extremotolerant rock inhabiting black fungi from Italian monumental sites. *Fungal Divers.* **2016**, *76*, 75–96, doi:10.1007/s13225-015-0342-9.
6. Crous, P.; Groenewald, J. Why everlastings don't last. *Persoonia—Mol. Phylogeny Evol. Fungi* **2011**, *26*, 70–84, doi:10.3767/003158511x574532.
7. de Hoog, G.; Vicente, V.; Najafzadeh, M.J.; Harrak, M.; Badali, H.; Seyedmousavi, S. Waterborne *Exophiala* species causing disease in cold-blooded animals. *Persoonia—Mol. Phylogeny Evol. Fungi* **2011**, *27*, 46–72, doi:10.3767/003158511x614258.
8. Bates, S.T.; Reddy, G.S.N.; Garcia-Pichel, F. *Exophiala crusticola* anam. nov. (affinity Herpotrichiellaceae), a novel black yeast from biological soil crusts in the Western United States. *Int. J. Syst. Evol. Microbiol.* **2006**, *56*, 2697–2702, doi:10.1099/ijs.0.64332-0.
9. Prenafeta-Boldú, F.X.; Summerbell, R.; de Hoog, G.S. Fungi growing on aromatic hydrocarbons: biotechnology's unexpected encounter with biohazard? *FEMS Microbiol. Rev.* **2006**, *30*, 109–130, doi:10.1111/j.1574-6976.2005.00007.x.
10. de Hoog, G.S.; Vicente, V.; Caligiorne, R.B.; Kantarcioglu, S.; Tintelnot, K.; Gerrits van den Ende, A.H.G.; Haase, G. Species diversity and polymorphism in the *Exophiala spinifera* clade containing opportunistic black yeast-like fungi. *J. Clin. Microbiol.* **2003**, *41*, 4767–4778, doi:10.1128/jcm.41.10.4767-4778.2003.
11. Isola, D.; Selbmann, L.; de Hoog, G.S.; Fenice, M.; Onofri, S.; Prenafeta-Boldú, F.X.; Zucconi, L. Isolation and screening of black fungi as degraders of volatile aromatic hydrocarbons. *Mycopathologia* **2013**, *175*, 369–379, doi:10.1007/s11046-013-9635-2.
12. Lian, X.; de Hoog, G.S. Indoor wet cells harbour melanized agents of cutaneous infection. *Med Mycol.* **2010**, *48*, 622–628, doi:10.3109/13693780903405774.
13. Seyedmousavi, S.; Badali, H.; Chlebicki, A.; Zhao, J.; Prenafeta-Boldú, F.X.; de Hoog, G.S. *Exophiala sideris*, a novel black yeast isolated from environments polluted with toxic alkyl benzenes and arsenic. *Fungal Biol.* **2011**, *115*, 1030–1037, doi:10.1016/j.funbio.2011.06.004.
14. de Hoog, G.S.; Zeng, J.S.; Harrak, M.J.; Sutton, D.A. *Exophiala xenobiotica* sp. nov., an opportunistic black yeast inhabiting environments rich in hydrocarbons. *Antonie van Leeuwenhoek* **2006**, *90*, 257–268, doi:10.1007/s10482-006-9080-z
15. Liu, X.-Y.; Udayanga, D.; Luo, Z.-L.; Chen, L.-J.; Zhou, D.-Q.; Su, H.Y.; Hyde, K.D. Backbone tree for Chaetothyriales with four new species of *Minimelanolocus* from aquatic habitats. *Fungal Biol.* **2015**, *119*, 1046–1062, doi:10.1016/j.funbio.2015.08.005.
16. Vigueras, G.; Arriaga, S.; Shirai, K.; Morales, M.; Revah, S. Hydrophobic response of the fungus *Rhinocladiella similis* in the biofiltration with volatile organic compounds with different polarity. *Biotechnol. Lett.* **2009**, *31*, 1203–1209, doi:10.1007/s10529-009-9987-3.
17. Hubka, V.; Réblová, M.; Řehulka, J.; Selbmann, L.; Isola, D.; de Hoog, S.G.; Kolarik, M. *Bradymyces* gen. nov. (Chaetothyriales, Trichomeriaceae), a new ascomycete genus accommodating poorly differentiated melanized fungi. *Antonie van Leeuwenhoek* **2014**, *106*, 979–992, doi:10.1007/s10482-014-0267-4.
18. Réblová, M.; Hubka, V.; Thureborn, O.; Lundberg, J.; Sallstedt, T.; Wedin, M.; Ivarsson, M. From the tunnels into the treetops: new lineages of black yeasts from biofilm in the Stockholm metro system and their relatives among ant-associated fungi in the Chaetothyriales. *PLoS ONE* **2016**, *11*, e0163396, doi:10.1371/journal.pone.0163396.
19. He, F.; Lin, B.; Sun, J.; Liu, X. *Knufia aspidiotus* sp. nov., a new black yeast from scale insects. *Phytotaxa* **2013**, *153*, 39–50, doi:10.11646/phytotaxa.153.1.2.
20. Hutchison, L.J.; Untereiner, W.A.; Hiratsuka, Y. *Knufia cryptophialidica* gen. et sp. nov., a dematiaceous hyphomycete isolated from black galls of trembling aspen (*Populus tremuloides*). *Mycologia* **1995**, *87*, 902–908, doi:10.1080/00275514.1995.12026613.
21. Tsuneda, A.; Currah, R.S. *Knufia endospora*, a new dematiaceous hyphomycete from trembling aspen. *Rep. Tottori. Mycol. Inst.* **2004**, *4*, 1–9.
22. Li, D.M.; de Hoog, G.S.; Saunte, D.M.L.; Gerrits van den Ende, A.H.G.; Chen, X.R. *Coniosporium epidermidis* sp. nov., a new species from human skin. *Stud. Mycol.* **2008**, *61*, 131–136, doi:10.3114/sim.2008.61.13.

23. Schiaparelli, S.; Selbmann, L.; Onofri, S.; Zucconi, L.; Isola, D.; Rottigni, M.; Ghiglione, C.; Piazza, P.; Alvaro, M.C. Distributional records of Antarctic fungi based on strains preserved in the Culture Collection of Fungi from Extreme Environments (CCFEE) Mycological Section associated with the Italian National Antarctic Museum (MNA). *MycoKeys* **2015**, *10*, 57–71, doi:10.3897/mycokeys.10.5343
24. Sun, W.; Su, L.; Yang, S.; Sun, J.; Liu, B.; Fu, R.; Wu, B.; Liu, X.; Cai, L.; Guo, L.; et al. Unveiling the hidden diversity of rock-inhabiting fungi: Chaetothyriales from China. *J. Fungi* **2020**, *6*, 187, doi:10.3390/jof6040187.
25. Mehrabi, M.; Asgari, B.; Hemmati, R. *Knufia perfecta*, a new black yeast from Iran, and a key to *Knufia* species. *Nova Hedwig.* **2018**, *106*, 519–534, doi:10.1127/nova_hedwigia/2017/0450.
26. Nai, C.; Wong, H.Y.; Pannenbecker, A.; Broughton, W.J.; Benoit, I.; de Vries, R.; Gueidan, C.; Gorbushina, A.A. Nutritional physiology of a rock-inhabiting, model microcolonial fungus from an ancestral lineage of the Chaetothyriales (Ascomycetes). *Fungal Genet. Biol.* **2013**, *56*, 54–66, doi:10.1016/j.fgb.2013.04.001.
27. Matos, T.; de Hoog, G.S.; de Boer, A.G.; de Crom, I.; Haase, G.M. High prevalence of the neurotrope *Exophiala dermatitidis* and related oligotrophic black yeasts in sauna facilities. *Mycoses* **2002**, *45*, 373–377, doi:10.1046/j.1439-0507.2002.00779.x.
28. Crous, P.; Wingfield, M.; Lombard, L.; Roets, F.; Swart, W.; Alvarado, P.; Carnegie, A.; Moreno, G.; Luangsa-Ard, J.; Thangavel, R.; et al. Fungal Planet description sheets: 951–1041. *Persoonia—Mol. Phylogeny Evol. Fungi* **2019**, *43*, 223–425, doi:10.3767/persoonia.2019.43.06.
29. Crous, P.W.; Schroers, H.-J.; Groenewald, J.Z.; Braun, U.; Schubert, K. *Metulocladosporiella* gen. nov. for the causal organism of Cladosporium speckle disease of banana. *Mycol. Res.* **2006**, *110*, 264–275, doi:10.1016/j.mycres.2005.10.003.
30. Chomnunti, P.; Bhat, D.J.; Jones, E.B.G.; Chukeatirote, E.; Bahkali, A.H.; Hyde, K.D. Trichomeriaceae, a new sooty mould family of Chaetothyriales. *Fungal Divers.* **2012**, *56*, 63–76, doi:10.1007/s13225-012-0197-2.
31. Egidi, E.; de Hoog, G.S.; Isola, D.; Onofri, S.; Quaedvlieg, W.; De Vries, M.; Verkley, G.J.M.; Stielow, J.B.; Zucconi, L.; Selbmann, L. Phylogeny and taxonomy of meristematic rock-inhabiting black fungi in the Dothideomycetes based on multi-locus phylogenies. *Fungal Divers.* **2014**, *65*, 127–165, doi:10.1007/s13225-013-0277-y.
32. Dugan, F.; Braun, U.; Groenewald, J.; Crous, P. Morphological plasticity in *Cladosporium sphaerospermum*. *Persoonia—Mol. Phylogeny Evol. Fungi* **2008**, *21*, 9–16, doi:10.3767/003158508x334389.
33. Bensch, K.; Groenewald, J.; Braun, U.; Dijksterhuis, J.; Yáñez-Morales, M.D.J.; Crous, P. Common but different: The expanding realm of *Cladosporium*. *Stud. Mycol.* **2015**, *82*, 23–74, doi:10.1016/j.simyco.2015.10.001.
34. Crous, P.; Shivas, R.; Wingfield, M.; Summerell, B.; Rossman, A.; Alves, J.; Adams, G.; Barreto, R.; Bell, A.; Coutinho, M.; et al. Fungal Planet description sheets: 128–153. *Persoonia—Mol. Phylogeny Evol. Fungi* **2012**, *29*, 146–201, doi:10.3767/003158512X661589.
35. Martin-Sanchez, P.M.; Novakova, A.; Bastian, F.; Alabouvette, C.; Saiz-Jimenez, C. Two new species of the genus *Ochroconis*, *O. lascauxensis* and *O. anomala* isolated from black stains in Lascaux Cave, France. *Fungal Biol.* **2012**, *116*, 574–589, doi:10.1016/j.funbio.2012.02.006.
36. Samerpitak, K.; Gerrits van den Ende, A.H.G.; Menken, S.B.J.; de Hoog, G.S. Three new species of the genus *Ochroconis*. *Mycopathologia* **2015**, *180*, 7–17, doi:10.1007/s11046-015-9910-5.
37. Crous, P.; Wingfield, B.; Burgess, T.; Hardy, G.; Crane, C.; Barrett, S.; Cano-Lira, J.F.; Le Roux, J.; Thangavel, R.; Guarro, J.; et al. Fungal Planet description sheets: 469–557. *Persoonia—Mol. Phylogeny Evol. Fungi* **2016**, *37*, 218–403, doi:10.3767/003158516x694499.
38. Giraldo, A.; Sutton, D.A.; Samerpitak, K.; de Hoog, G.S.; Wiederhold, N.; Guarro, J.; Gené, J. Occurrence of *Ochroconis* and *Verruconis* species in clinical specimens from the United States. *J. Clin. Microbiol.* **2014**, *52*, 4189–4201, doi:10.1128/jcm.02027-14.
39. Crous, P.; Wingfield, M.; Richardson, D.; Le Roux, J.; Strasberg, D.; Edwards, J.; Roets, F.; Hubka, V.; Taylor, P.; Heykoop, M.; et al. Fungal Planet description sheets: 400–468. *Persoonia—Mol. Phylogeny Evol. Fungi* **2016**, *36*, 316–458, doi:10.3767/003158516x692185.
40. Samerpitak, K.; Duarte, A.P.M.; Attili-Angelis, D.; Pagnocca, F.C.; Heinrichs, G.; Rijs, A.J.M.M.; Alfjorden, A.; Gerrits van den Ende, A.H.G.; Menken, S.B.J.; de Hoog, G.S. A new species of the oligotrophic genus *Ochroconis* (Sympoventuriaceae). *Mycol. Prog.* **2015**, *14*, 1–10, doi:10.1007/s11557-015-1023-5.
41. Samerpitak, K.; Van der Linde, E.; Choi, H.-J.; Gerrits van den Ende, A.H.G.; Machouart, M.; Gueidan, C.; de Hoog, G.S. Taxonomy of *Ochroconis*, genus including opportunistic pathogens on humans and animals. *Fungal Divers.* **2014**, *65*, 89–126, doi:10.1007/s13225-013-0253-6.
42. Crous, P.W.; Shivas, R.; Quaedvlieg, W.; Van Der Bank, M.; Zhang, Y.; Summerell, B.; Guarro, J.; Wingfield, M.J.; Wood, A.; Alfenas, A.; et al. Fungal Planet description sheets: 214–280. *Persoonia—Mol. Phylogeny Evol. Fungi* **2014**, *32*, 184–306, doi:10.3767/003158514x682395.
43. Crous, P.W.; Wingfield, M.J.; Le Roux, J.J.; Richardson, D.M.; Strasberg, D.; Shivas, R.G.; Groenewald, J.Z. Fungal Planet description sheets: 371–399. *Persoonia* **2015**, *35*, 264, doi:10.3767/003158515X69026.
44. Jayasiri, S. Diversity, morphology and molecular phylogeny of Dothideomycetes on decaying wild seed pods and fruits. *Mycosphere* **2019**, *10*, 1–186, doi:10.5943/mycosphere/10/1/1.
45. Machouart, M.; Samerpitak, K.; de Hoog, G.S.; Gueidan, C. A multigene phylogeny reveals that *Ochroconis* belongs to the family Sympoventuriaceae (Venturiales, Dothideomycetes). Fungal Divers. **2014**, *65*, 77–88, doi:10.1007/s13225-013-0252-7
