# Supplementary figures and images for: Hydrocarbon-Contaminated Sites: Is There Something More Than Exophiala xenobiotica? New Insights into Black Fungal Diversity Using the Long Cold Incubation Method
Source: J Fungi (Basel). 2021 Sep 29;7(10):817. doi: 10.3390/jof7100817 (PMC8538888; doi:10.3390/jof7100817)

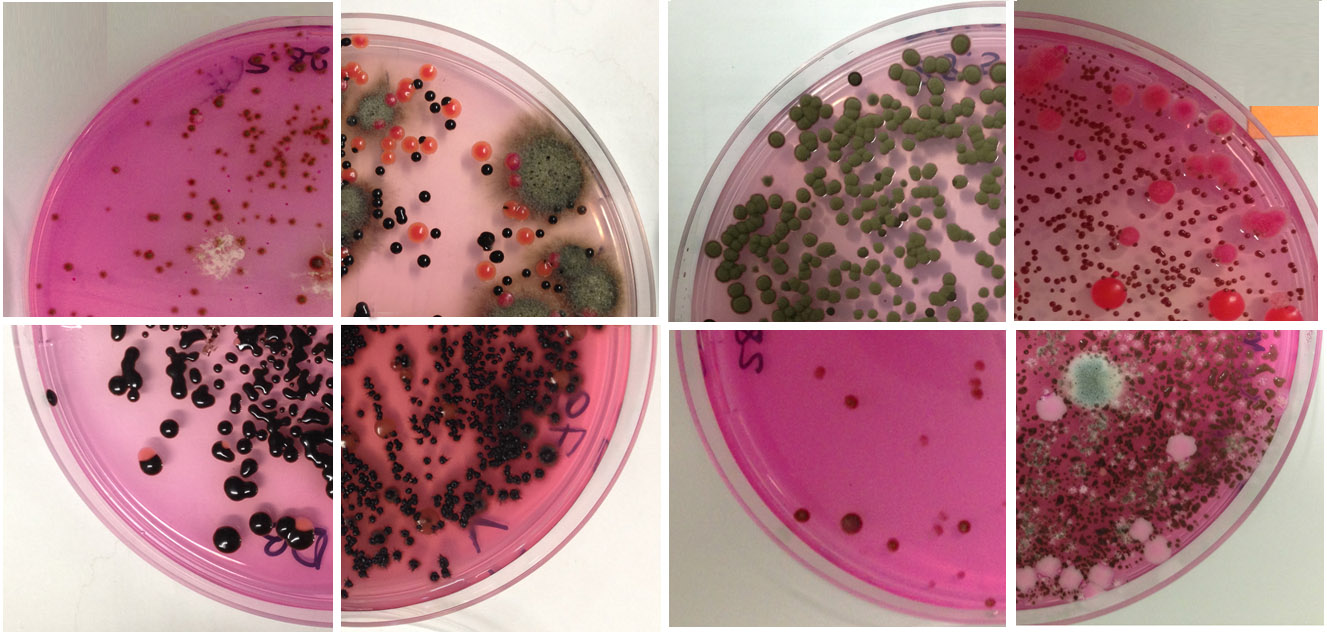

Supplement: Supplementary file 1 [file jof-07-00817-s001.zip › jof-1326457/Fig_S1.jpg]

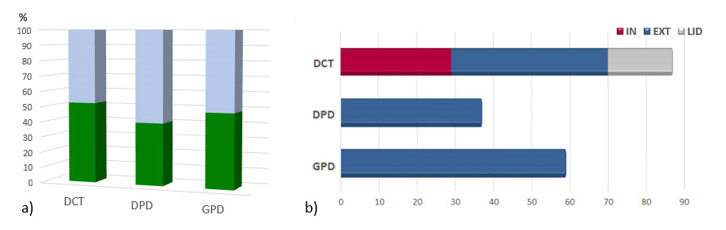

Supplement: Supplementary file 1 [file jof-07-00817-s001.zip › jof-1326457/Fig_S2.jpg]

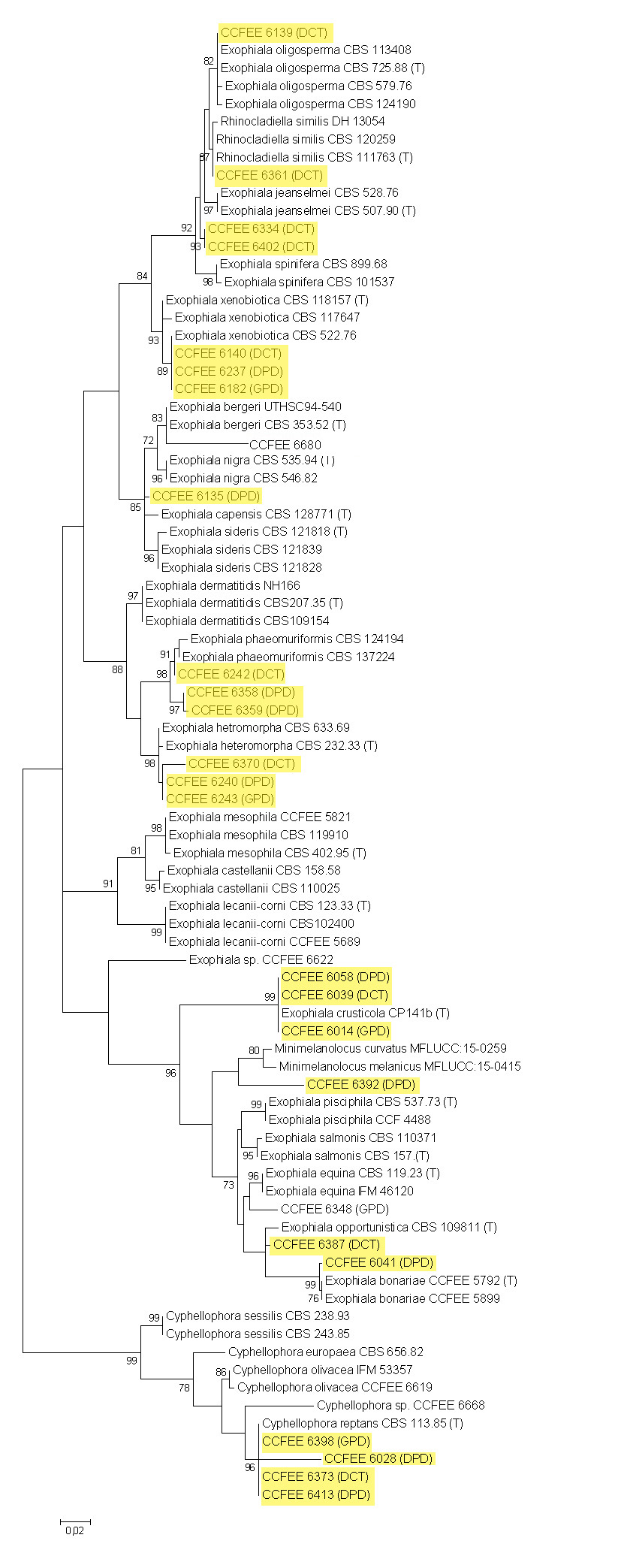

Supplement: Supplementary file 1 [file jof-07-00817-s001.zip › jof-1326457/FiG_S3.jpg]

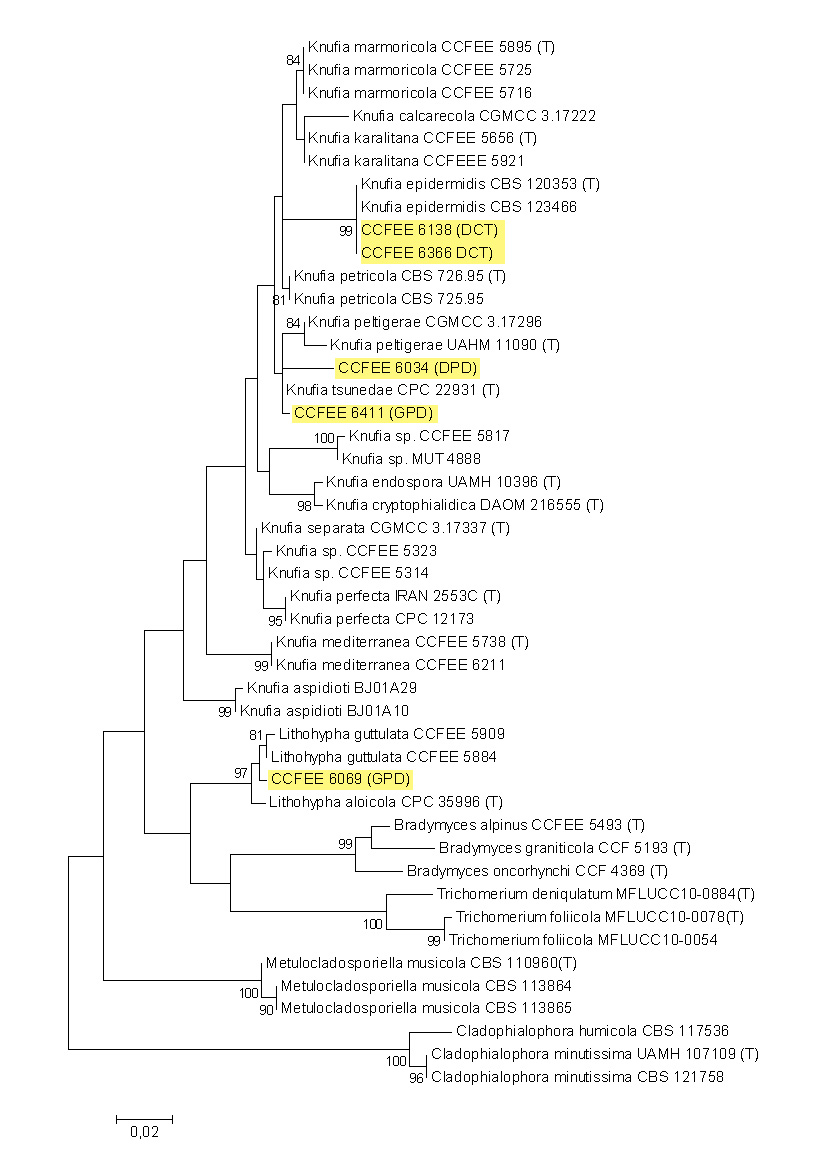

Supplement: Supplementary file 1 [file jof-07-00817-s001.zip › jof-1326457/Fig_S4.jpg]

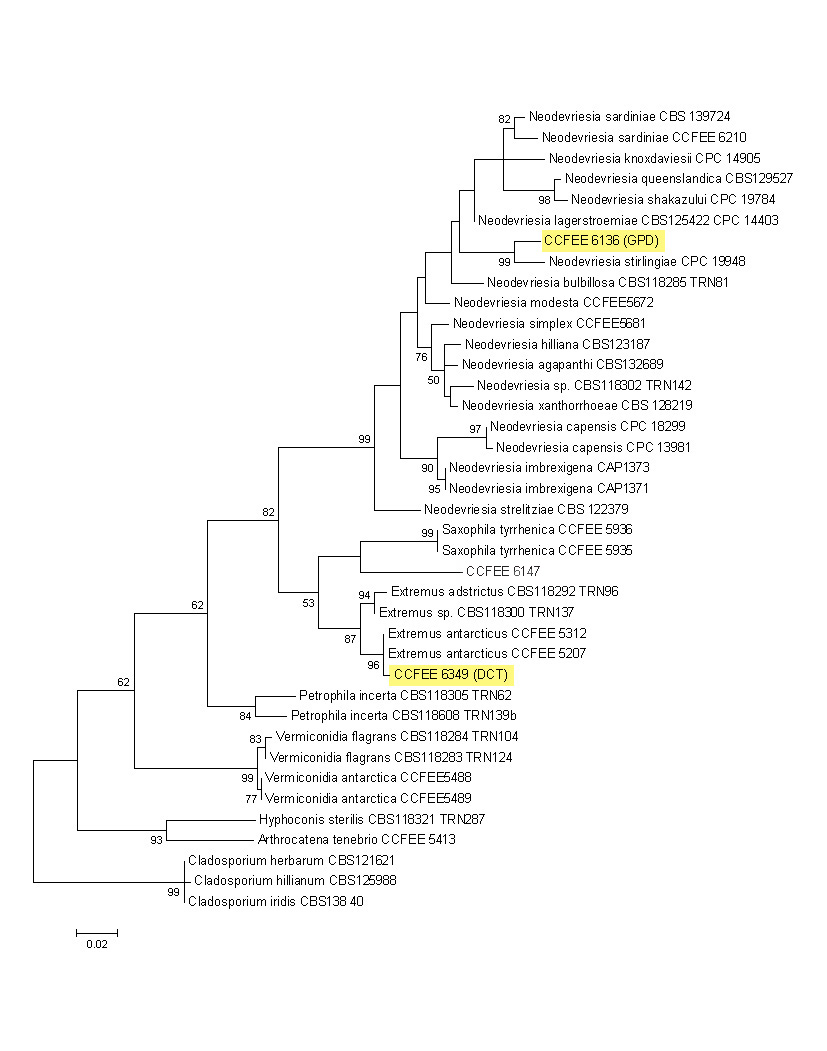

Supplement: Supplementary file 1 [file jof-07-00817-s001.zip › jof-1326457/Fig_S5.jpg]

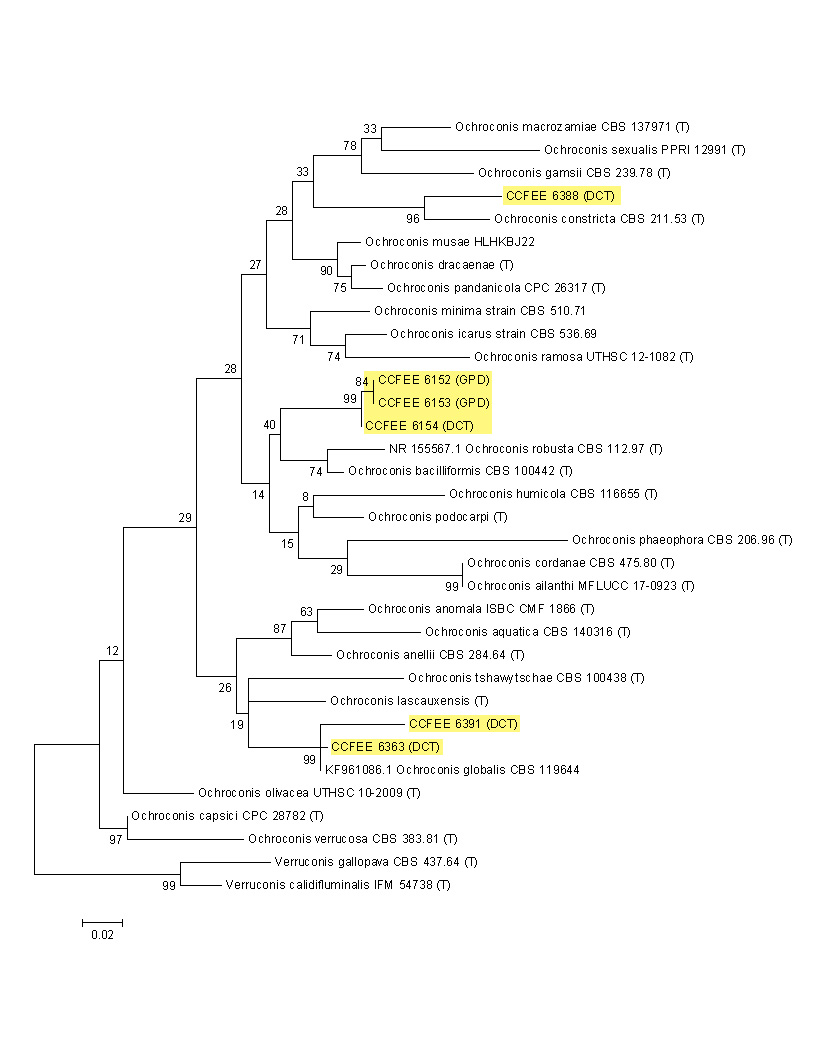

Supplement: Supplementary file 1 [file jof-07-00817-s001.zip › jof-1326457/Fig_S6.jpg]

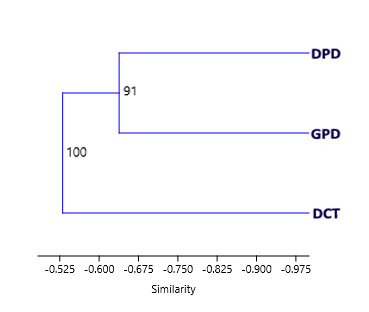

Supplement: Supplementary file 1 [file jof-07-00817-s001.zip › jof-1326457/Fig_S7.jpg]

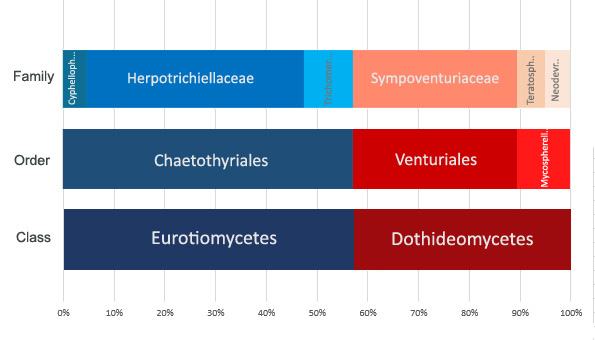

Supplement: Supplementary file 1 [file jof-07-00817-s001.zip › jof-1326457/Fig_S8.jpg]
